# Carbon Hollow Fiber Penetration Electrode with Unsaturated Ni‐N_2_ Coordination for Enhanced CO_2_ Electroreduction

**DOI:** 10.1002/advs.202502947

**Published:** 2025-05-20

**Authors:** Xiaotong Wang, Yiheng Wei, Yanfang Song, Jianing Mao, Xiaohu Liu, Shoujie Li, Guihua Li, Huanyi Zhu, Jiayu Xia, Cheng Luo, Aohui Chen, Xiao Dong, Wei Wei, Wei Chen

**Affiliations:** ^1^ Low‐Carbon Conversion Science and Engineering Center Shanghai Advanced Research Institute Chinese Academy of Sciences Shanghai 201210 P. R. China; ^2^ University of Chinese Academy of Sciences Beijing 100049 P. R. China; ^3^ State Key Laboratory of Low Carbon Catalysis and Carbon Dioxide Utilization Shanghai Advanced Research Institute Chinese Academy of Sciences Shanghai 201210 P. R. China; ^4^ Shanghai Institute of Applied Physics Chinese Academy of Sciences Shanghai 201204 P. R. China; ^5^ School of Physical Science and Technology ShanghaiTech University Shanghai 201203 P. R. China

**Keywords:** CO_2_ electroreduction, carbon hollow fiber, gas penetration electrode, Ni‐N‐C, unsaturated coordination

## Abstract

Hollow fiber gas penetration electrodes with a compact hierarchical pore structure have emerged as promising platforms for CO_2_ electroreduction. However, developing carbon hollow fiber electrodes with efficient CO_2_ electrocatalytic performance remains unexplored and challenging. Herein, a straightforward strategy is presented to fabricate robust, self‐standing carbon hollow fiber electrodes modified with an unsaturated Ni‐N_2_ coordination structure. This unique hollow fiber electrode configuration effectively enhances the kinetics of CO_2_ electro‐conversion to CO. Both density functional theory (DFT) calculations and experimental studies reveal that the Ni‐N_2_ structure significantly boosts electrocatalytic activity for CO_2_ reduction by reducing the energy barrier for the key intermediate COOH* formation. Consequently, the electrode with unsaturated Ni‐N_2_ coordination realizes a high CO Faradaic efficiency (FE) (>90%) as well as a partial current density of 61 mA cm^−2^, much superior to those of saturated Ni‐N_4_ coordination. In particular, this high performance maintains an exceptional durability for over 100 h, outperforming previously reported carbon supporting electrodes featuring Ni‐N‐C sites. This work opens new avenues for designing advanced carbon electrode structures with enhanced selectivity and activity for CO_2_ reduction.

## Graphical abstract

A hierarchical porous carbon hollow fiber anchoring Ni‐N_2_ structures is developed as a self‐supporting electrode for efficient and stable CO_2_ conversion to CO. The unique penetration effect of the hollow fiber electrode, combined with the unsaturated Ni‐N_2_ coordination structure, significantly enhances CO_2_ electroreduction performance, achieving high activity and long‐term durability.

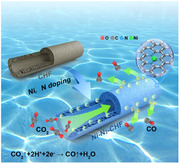

## 1 Introduction

Excessive CO_2_ emissions have led to severe environmental challenges, threatening sustainable development.^[^ ^1^, ^2^ ^]^ Electrocatalytic CO_2_ reduction reaction (ECO_2_RR) powered by renewable electricity offers a viable pathway to convert CO_2_ into valuable fuels and chemicals, which can effectively realize negative carbon cycling. However, this process faces several bottlenecks, including the limited solubility of CO_2_ in electrolytes, sluggish reaction kinetics as well as competition with the hydrogen evolution reaction (HER), which complicates achieving high selectivity at elevated current densities.^[^ ^3^, ^4^, ^5^, ^6^ ^]^ Hollow fiber gas penetration electrodes have shown great potential in addressing these challenges due to their unique structural attributes. Their three‐dimensional, compact, hierarchical pore structure can compel CO_2_ to penetrate through the porous walls, ensuring efficient contact between CO_2_, catalysts, and electrolytes, which improves mass transfer and three‐phase boundary reactions.^[^ ^7^, ^8^ ^]^ To date, advances in metallic hollow fiber electrodes have demonstrated exceptional ECO_2_RR performance.^[^ ^7^, ^9^, ^10^, ^11^, ^12^, ^13^, ^14^ ^]^ However, developing carbon hollow fiber electrodes for efficient ECO_2_RR remains unexplored and poses significant challenges due to their difficult preparation and catalytical inertia.

Carbon‐based electrocatalysts are promising for ECO_2_RR owing to their abundance, stability, and tunable structure.^[^ ^15^, ^16^, ^17^ ^]^ Introducing heteroatoms like nitrogen (N), phosphorus (P), or sulfur (S) into the carbon networks can alter the charge density and transform the inert carbon structure to be active for CO_2_ adsorption and conversion.^[^ ^18^, ^19^, ^20^, ^21^, ^22^ ^]^ Additionally, carbon electrodes can also serve as excellent carriers for anchoring single‐metal atoms through nitrogen coordination, which is conducive to increasing active sites and enhancing catalytic activity.^[^ ^22^, ^23^, ^24^, ^25^ ^]^ Recent studies suggest that unsaturated metal coordination can further boost ECO_2_RR performance by promoting CO_2_ activation and lowering the energy barrier for intermediate formation.^[^ ^26^, ^27^, ^28^, ^29^, ^30^, ^31^ ^]^ However, precisely regulating the unsaturated coordination structures, particularly on the curved surface of self‐standing porous carbon electrodes, remains a significant challenge.

Herein, we explored a robust, self‐standing carbon hollow fiber electrode tailored with unsaturated Ni‐N_2_ coordinations (donated as Ni‐N_2_‐CHF), which dramatically boosts CO_2_ electroreducion to CO. The hollow fiber penetration electrode structure enhances mass transfer and three‐phase interfacial reactions during electrolysis. Furthermore, the unsaturated Ni‐N_2_ coordination with symbiotic Ni_2_ clusters greatly lowers the energy barrier for the key intermediate *COOH formation. As a result, the Ni‐N_2_‐CHF electrode achieves a FE of 91.0% and excellent durability over 100 h for CO generation at −1.0 V versus the reversible hydrogen electrode (vs. RHE) in 0.5 M KHCO_3_. These metrics significantly surpass those of electrodes with saturated Ni‐N_4_ coordination. Our findings provide new insights into designing self‐standing carbon electrodes with advanced active sites, offering significant potential for sustainable CO_2_ electroreduction technologies.

## 2 Results and Discussion

**Figure 1:**
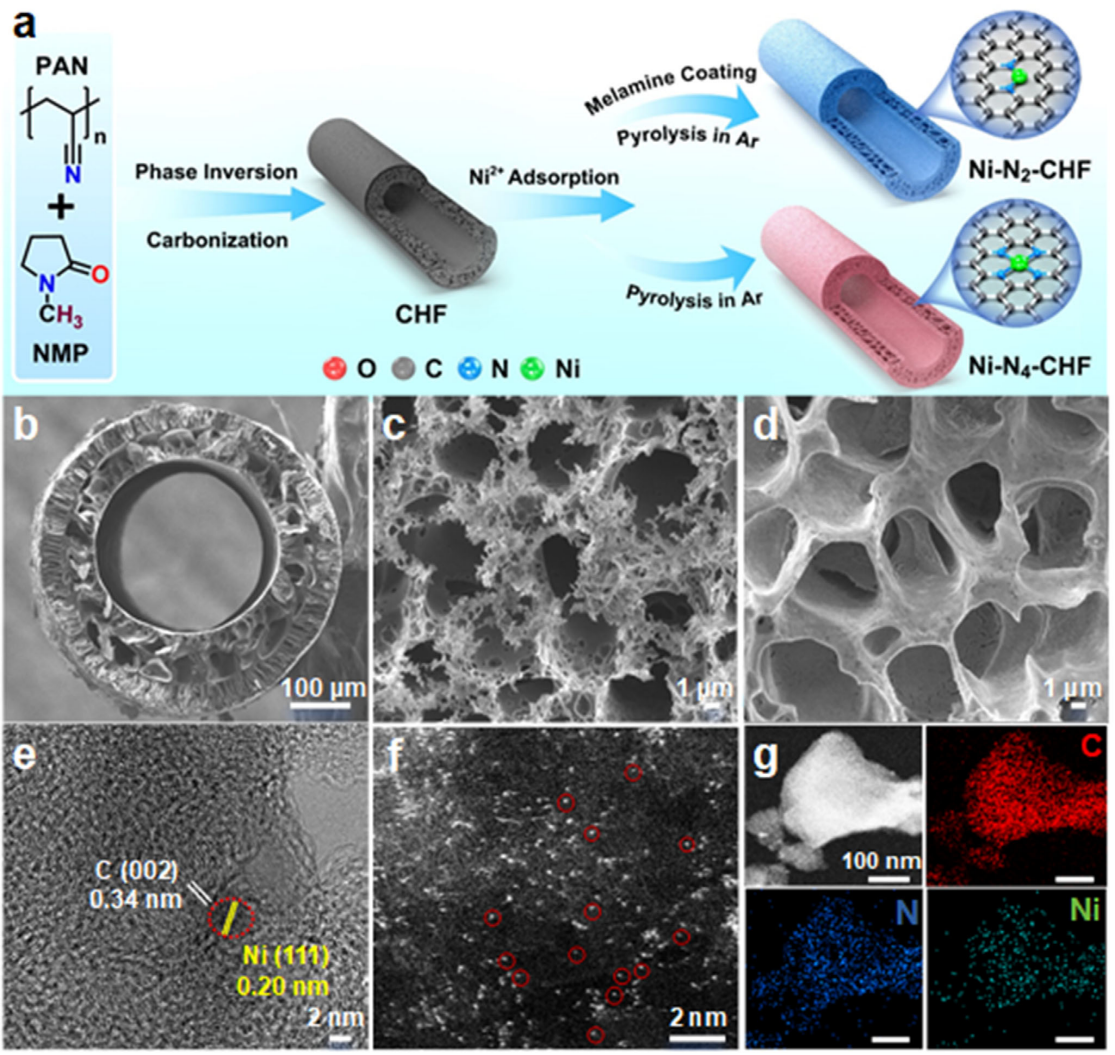
a) Schematic illustration of Ni‐N_2_‐CHF preparation. b) SEM image for the cross‐section of Ni‐N_2_‐CHF. SEM images for the outer surfaces of c) NCHF and d) Ni‐N_2_‐CHF. e) High‐resolution TEM image for Ni‐N_2_‐CHF. f) HAADF‐STEM image for Ni‐N_2_‐CHF showing Ni single atoms (red circles) and g) the corresponding element mappings for Ni‐N_2_‐CHF.

The carbon hollow fiber (CHF) was first fabricated using phase inversion and pyrolysis methods. Subsequent Ni, N co‐doping yielded the Ni‐N_2_‐CHF electrode (**Figure** **1**a; Figure S1, Supporting Information). The Ni‐N_2_ coordination environment arises from the coordinative reconstruction of Ni single atoms facilitated by a facile melamine‐assisted pyrolysis strategy. For comparison, we also prepared N‐doped carbon hollow fiber (NCHF) and Ni‐doped carbon hollow fiber with a saturated Ni‐N_4_ configuration (Ni‐N_4_‐CHF) to elucidate the superiority of the unsaturated Ni‐N_2_ configuration.

Scanning electron microscopy (SEM) confirms the hierarchical porous structure of Ni‐N_2_‐CHF, with a diameter of ≈460 µm and a wall thickness of ≈120 µm. The cross‐sectional view reveals finger‐like pores, while the outer surface displays a honeycomb‐like structure, that is more porous than NCHF, attributed to Ni incorporation (Figure 1b‐d; Figure S2, Supporting Information). Elemental mapping displays uniform distribution of C, N as well as Ni without detectable large Ni particles (Figure S3, Supporting Information). High‐resolution transmission electron microscopy (HRTEM) images exhibit distinct lattice fringes with spacings of 0.34 nm and 0.20 nm (**Figur** **e** 1e), corresponding to the (002) plane of graphitic carbon and the (111) plane of metallic Ni, respectively. Encapsulated Ni nanoparticles within the carbon layer of Ni‐N_2_‐CHF and Ni‐N_4_‐CHF are ≈3.3 and 5.5 nm in size, respectively (Figure S4, Supporting Information). These small particle sizes, along with the thick carbon layers, were reported to have negligible effects on catalytic performance.^[^ ^32^, ^33^, ^34^, ^35^ ^]^

High‐angle annular dark‐field scanning transmission electron microscopy (HAADF‐STEM) image at atomic resolution displays numerous bright spots on both Ni‐N_2_‐CHF and Ni‐N_4_‐CHF (Figure 1f; Figure S5a, Supporting Information), indicating the presence of highly dispersed isolated Ni single atoms (highlighted by red circles) and multinuclear Ni nanoclusters. Corresponding elemental mappings confirm the uniform distribution of C, N as well as Ni on Ni‐N_2_‐CHF and Ni‐N_4_‐CHF (Figure 1g; Figure S5b, Supporting Information). The Ni content in Ni‐N_2_‐CHF and Ni‐N_4_‐CHF is determined through inductively coupled plasma optical emission spectroscopy (ICP‐OES) to be 3.66 wt.% and 3.39 wt.%, respectively. (Table S1, Supporting Information).

Raman spectra display two characteristic peaks at 1363 cm^−1^ and 1576 cm^−1^, corresponding to the D and G bands of carbon materials, respectively (Figure S6a, Supporting Information).^[^ ^36^ ^]^ The Ni‐N_2_‐CHF sample exhibits an increased intensity ratio of D to G bands (I_D_/I_G_) compared to Ni‐N_4_‐CHF, NCHF, and CHF, indicating that co‐doping with Ni and N introduces additional defects into the carbon structure. The higher gas permeability of Ni‐N_2_‐CHF (Figure S6b, Supporting Information) corroborates the formation of defects or pores, consistent with the SEM observations.

**Figure 2:**
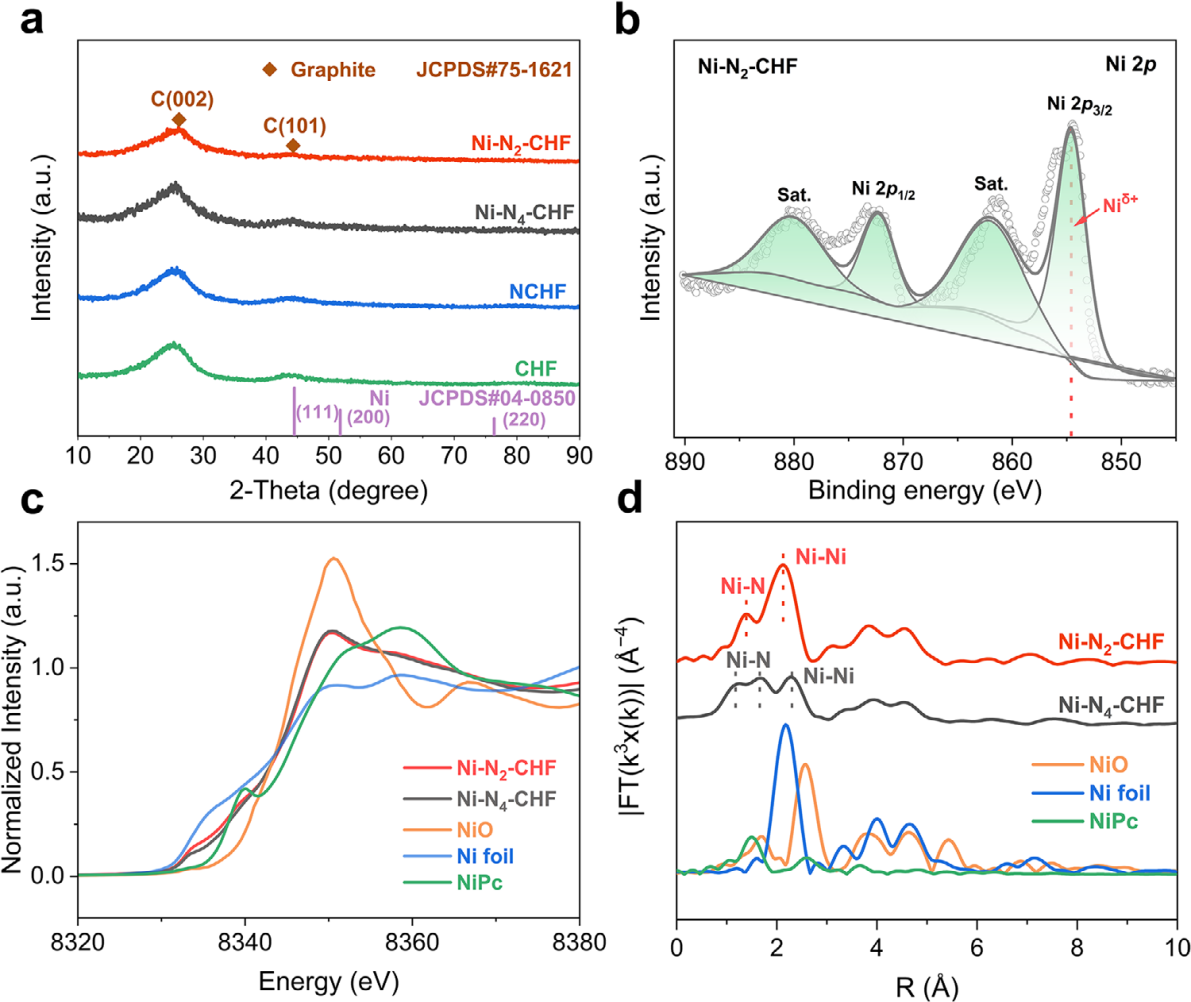
a) XRD patterns of CHF, NCHF, Ni‐N_2_‐CHF, and Ni‐N_4_‐CHF. b) Ni 2p XPS of Ni‐N_2_‐CHF. c) Ni K‐edge XANES spectra and d) FT‐EXAFS spectra of Ni‐N_2_‐CHF and Ni‐N_4_‐CHF with NiO, Ni foil, and NiPc as references.

X‐ray Diffraction (XRD) patterns for all samples exhibit two broad diffraction peaks at 26.1° and 44.3° (**Figure** **2**a), which are attributed to the (002) and (101) crystal planes of graphitic carbon. The absence of diffraction peaks connected with crystalline Ni species, suggests no detectable Ni nanoparticles, further confirming the encapsulation within a thick carbon layer. To study the chemical composition and electronic states on the electrode surfaces, X‐ray photoelectron spectroscopy (XPS) analyses were conducted (Figure S7, Supporting Information). The Ni 2p spectra of Ni‐N_2_‐CHF and Ni‐N_4_‐CHF show Ni 2p_3/2_ peaks at 854.6 eV and 855.6 eV, respectively (Figure 2b; Figure S7b, Supporting Information). These peaks, positioned between those for Ni^0^ (853.5 eV) and Ni^2+^ (856.0 eV), suggest the presence of Ni^δ+^ centers (0<δ<2) in both Ni‐N_2_‐CHF and Ni‐N_4_‐CHF.^[^ ^32^, ^37^ ^]^ The nitrogen concentration and atomic configurations are further analyzed. The N 1s spectra of Ni‐N_2_‐CHF and Ni‐N_4_‐CHF are deconvoluted into components corresponding to pyridinic N (398.3 eV), Ni‐N (399.2 eV), pyrrolic N (400.3 eV), graphitic N (401.2 eV), and oxidized N (402.8 eV) (Figure S7, Supporting Information), which indicates the formation of the Ni‐N‐C structure. The atomic concentrations of pyridinic N, Ni‐N, and pyrrolic N in Ni‐N_2_‐CHF are 2.31, 1.30, and 2.41 at%, respectively (Table S2, Supporting Information), compared to 1.10, 1.31, and 0.83 at.% in Ni‐N_4_‐CHF, demonstrating that post‐N‐doping enhances the incorporation of nitrogen reactive species.

The electronic structure and coordination environment of Ni species in Ni‐N_2_‐CHF and Ni‐N_4_‐CHF were further investigated using X‐ray adsorption near‐edge structure (XANES) spectroscopy and extended X‐ray adsorption fine structure (EXAFS) spectroscopy. Ni foil, NiO, and NiPc were used as references for Ni^0^, Ni^2+^, and Ni‐N, respectively. As displayed in Figure 2c, the XANES spectra for Ni‐N_2_‐CHF and Ni‐N_4_‐CHF exhibit pre‐edge energy positions between those of NiO and Ni foil, revealing that the Ni atoms possess positive valence states of Ni^δ+^ (0 < δ < 2), consistent with the XPS results. Furthermore, the oxidation states of Ni in Ni‐N_2_‐CHF and Ni‐N_4_‐CHF, determined from XANES edge energies, are +0.46 and +0.78, respectively (Table S3, Supporting Information), revealing a lower valence state for Ni in Ni‐N_2_‐CHF. The coordination environment of Ni in Ni‐N_2_‐CHF and Ni‐N_4_‐CHF was probed by Fourier‐transformed extended X‐ray absorption fine structure (FT‐EXAFS). For Ni‐N_2_‐CHF, prominent peaks at 1.38 and 2.13 Å correspond to Ni‐N and Ni‐Ni coordination shells, respectively (Figure 2d). In contrast, the Ni‐N_4_‐CHF displays peaks at 1.18 and 1.66 Å for the Ni‐N shell and 2.29 Å for the Ni‐Ni shell. The reduced intensity and slight shift in the Ni‐Ni peak for both Ni‐N_2_‐CHF and Ni‐N_4_‐CHF compared to Ni foil demonstrate the existence of Ni nanoclusters. EXAFS fitting analyses reveal Ni‐N coordination numbers of 1.9 and 3.9 for Ni‐N_2_‐CHF and Ni‐N_4_‐CHF, respectively, corresponding to Ni‐N_2_ and Ni‐N_4_ configurations. The Ni‐Ni coordination numbers are 2.3 and 2.4 for Ni‐N_2_‐CHF and Ni‐N_4_‐CHF, respectively (Figure S8 and Table S4, Supporting Information), demonstrating the presence of Ni nanoclusters.^[^ ^38^, ^39^, ^40^ ^]^ Wavelet‐transformed (WT) EXAFS contour patterns further verify the coexistence of the Ni‐N as well as Ni‐Ni coordination pairs in both Ni‐N_2_‐CHF and Ni‐N_4_‐CHF (Figure S9, Supporting Information).^[^ ^41^ ^]^

The electron paramagnetic resonance (EPR) spectra of all samples show a *g*‐value of 2.005 (Figure S10, Supporting Information), which is ascribed to unpaired electrons trapped at nitrogen vacancies.^[^ ^42^ ^]^ Compared to the Ni‐N_4_‐CHF, Ni‐N_2_‐CHF exhibits a relatively higher spin concentration (Table S5, Supporting Information), suggesting a greater abundance of nitrogen vacancies in the carbon framework. Similarly, NCHF contains more nitrogen vacancies than CHF, which likely facilitates the generation of unsaturated Ni‐N_2_ coordination when Ni atoms are incorporated.

The as‐prepared hollow fiber electrodes, as the self‐supporting working electrodes, were tested for CO_2_ electroreduction reaction in a homemade electrolytic cell (Figure S11, Supporting Information). Gaseous CO_2_ was injected into the interior of the hollow fiber electrodes and dispersed uniformly through the porous walls of the hollow fiber. The emerging bubbles (Figure S11c, Supporting Information) disturbed the electrolyte solution near the electrode, enhancing solution exchange with the bulk electrolyte. The ECO_2_RR performance was initially assessed by linear sweep voltammetry (LSV) under a CO_2_ atmosphere and compared to that in Ar (Figure S12, Supporting Information). The current densities of Ni‐N_2_‐CHF, Ni‐N_4_‐CHF, and NCHF electrodes in CO_2_ are significantly higher than those in Ar, suggesting the occurrence of ECO_2_RR. In contrast, the nearly overlapping LSV curves for CHF in CO_2_ and Ar indicate its sluggish activity for ECO_2_RR. Notably, Ni‐N_2_‐CHF achieves a much higher current density as well as a more positive onset potential than Ni‐N_4_‐CHF (Figure S12e, Supporting Information), implying that N‐vacancy‐rich unsaturated Ni‐N_2_ coordination substantially improves CO_2_ electrocatalytic performance.

Potentiostatic electrolysis of CO_2_ was subsequently performed using the self‐supporting hollow fiber electrodes in an aqueous KHCO_3_ electrolyte with a CO_2_ flow rate of 10 sccm. CO_2_ molecules penetrated the porous walls of the hollow fiber electrode, reacting with water at the active sites to produce CO. The electrocatalytic performance of Ni‐N_2_‐CHF was evaluated across KHCO_3_ concentrations ranging from 0.1 to 1 M, with optimal performance achieved at 0.5 M (Figure S13, Supporting Information). Consequently, the ECO_2_RR performance and electrochemical characteristics of all electrodes were evaluated in 0.5 M KHCO_3_.

**Figure 3:**
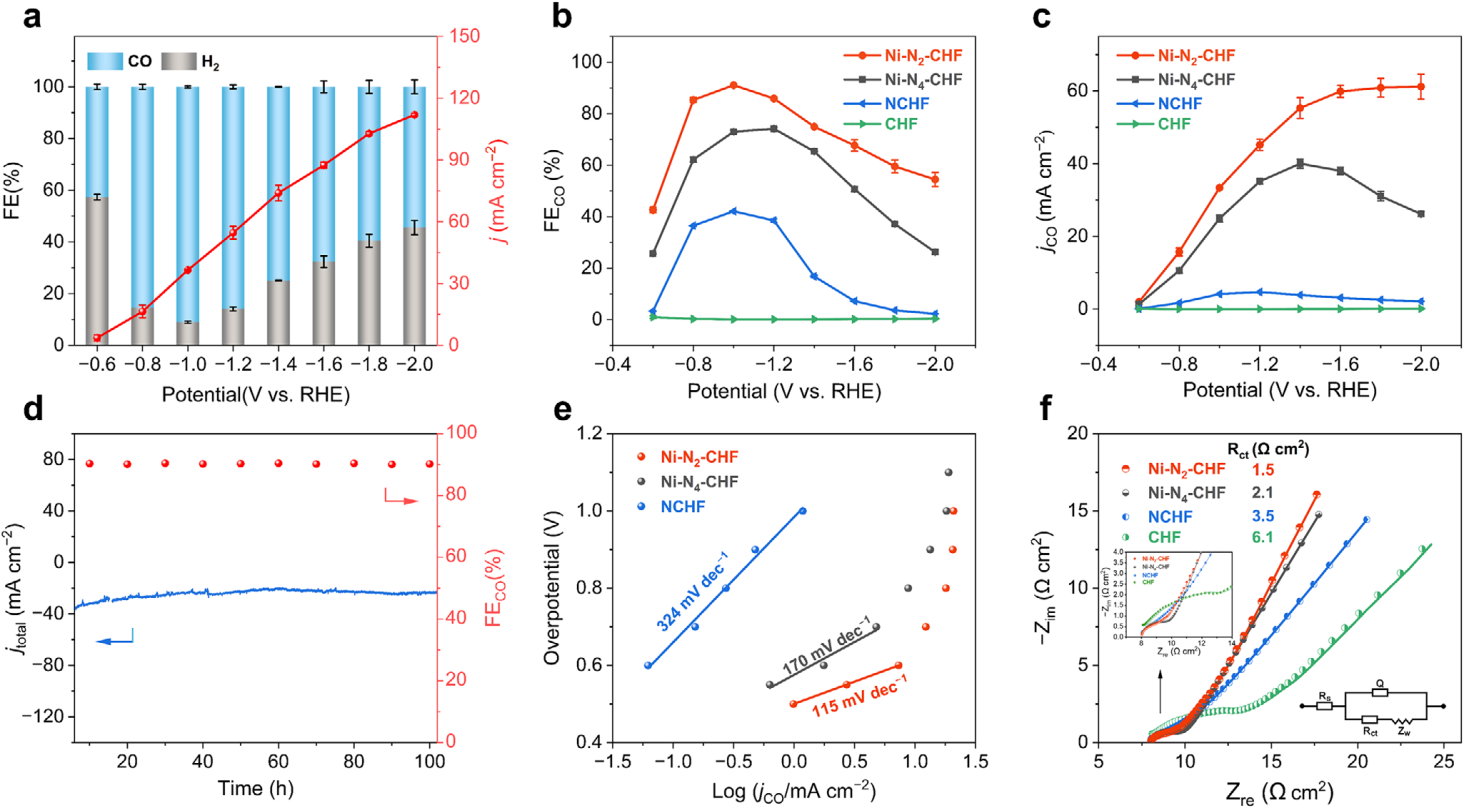
a) FEs of ECO_2_RR products on Ni‐N_2_‐CHF electrode under various potentials. b) The CO FE at various cathodic potentials over Ni‐N_2_‐CHF, Ni‐N_4_‐CHF, NCHF, and CHF. c) CO partial current densities of Ni‐N_2_‐CHF, Ni‐N_4_‐CHF, NCHF, and CHF at various cathodic potentials. d) Long‐term stability of Ni‐N_2_‐CHF at a potential of −1.0 V vs. RHE. e) Tafel slope plots of different electrodes toward CO production. f) EIS Nyquist plots at open circuit potential. Error bars were obtained from the standard deviation of three independent measurements.

Only CO and H_2_ are detected for the Ni‐N_2_‐CHF electrode during CO_2_ electrolysis over the potential range of −0.6 to −2.0 V vs. RHE, with their FE summing to nearly 100%. No liquid products are observed by ^1^H NMR spectroscopy (Figures S14, S15, Supporting Information). Distinctively, Ni‐N_2_‐CHF primarily produces CO, achieving FEs between 85% and 91% within a potential range from −0.8 to −1.2 V vs. RHE (**Figure** **3**a), with a maximum CO FE (FE_CO_) of 91.0% at −1.0 V vs. RHE. As further increasing the cathodic potential, HER becomes dominant, leading to a gradual decline in CO FE. Thus, the CO‐to‐H_2_ ratio can be tuned from 10:1 to 3:2, aligning with syngas requirements for various chemical processes. In contrast, the Ni‐N_4_‐CHF electrode, characterized by saturated Ni‐N_4_ coordination, delivers a maximum FE_CO_ of 73.7% at −1.2 V vs. RHE (Figure 3b). The NCHF electrode without Ni‐doping displays only 42.1% of FE_CO_ at −1.0 V vs. RHE, while the unmodified CHF electrode predominantly produces H_2_, with FE_CO_ consistently below 1.0% across all potentials. Additionally, upon a negative shift in potential, Ni‐N_2_‐CHF presents a sharp increase of CO partial current density (*j*_CO_) initially and then levels off, remaining at about 61 mA cm^−2^ between −1.6 and −2.0 V vs. RHE (Figure 3c). The maximum *j*_CO_ value of Ni‐N_2_‐CHF is approximately 1.5, 13, and 635 times higher than those of Ni‐N_4_‐CHF, NCHF, and CHF, respectively. Across all potentials, Ni‐N_2_‐CHF consistently shows superior FE_CO_ and *j*_CO_ performance compared to other electrodes (Figure 3b,c), underscoring its exceptional activity for ECO_2_RR. Additionally, NCHF presents a lower charge transfer resistance (*R*_ct_) than CHF due to the more abundant N‐active sites created by additional N‐doping. These contribute to the higher performance of NCHF compared to CHF (Figure 3b,c).

To investigate the origin of this superior performance of Ni‐N_2_‐CHF, the electrochemical active surface areas (ECSAs) of the prepared electrodes were assessed using the double‐layer capacitance (*C*_dl_) (Figure S16, Supporting Information). The Ni‐N_2_‐CHF exhibits the highest *C*_dl_ (1.75 mF cm^−2^), surpassing Ni‐N_4_‐CHF (1.40 mF cm^−2^), NCHF (0.69 mF cm^−2^), and CHF (0.50 mF cm^−2^), suggesting enhanced exposure of active sites due to increased defects. The CO partial current densities of the electrodes were subsequently normalized using ECSA. The results reveal that the Ni‐N_2_‐CHF possesses the highest ECSA‐normalized *j*_CO_ in the potential range from −0.6 to −1.6 V vs. RHE (Figure S17, Supporting Information). This finding confirms the superior intrinsic activity of the Ni‐N_2_‐CHF electrode for CO production. Contact angle measurements show a hydrophobicity trend of Ni‐N_2_‐CHF>Ni‐N_4_‐CHF>NCHF>CHF (Figure S18, Supporting Information), consistent with their ECO_2_RR performance. This suggests that the enhanced surface hydrophobicity of hollow fiber electrodes appears to favor ECO_2_RR. Reaction kinetics analysis via Tafel plots shows a slope of 115 mV dec^−1^ for Ni‐N_2_‐CHF, close to 118 mV dec^−1^, displaying that the first electron‐proton coupling step to form the *COOH intermediate is rate‐determining.^[^ ^43^ ^]^ In comparison, Tafel slopes for Ni‐N_4_‐CHF and NCHF are higher (170 mV dec^−1^ and 324 mV dec^−1^, respectively, Figure 3e), suggesting slower CO_2_ reduction kinetics. Electrochemical impedance spectroscopy (EIS) further corroborates the faster electron as well as mass transfer kinetics of Ni‐N_2_‐CHF, showing significantly lower charge transfer resistance (*R*_ct_) compared to the Ni‐N_4_‐CHF and NCHF (Figure 3f).

Long‐term stability measurements for Ni‐N_2_‐CHF conducted at −1.0 V vs. RHE reveal a sustained FE_CO_ above 90% during 100 h of electrolysis (Figure 3d), outperforming previously reported carbon supporting electrodes with Ni‐N‐C sites (Table S6, Supporting Information). The composition and electronic structure of Ni‐N_2_‐CHF after the long‐term ECO_2_RR test were further investigated. Post‐electrolysis analyses via XRD (Figure S19, Supporting Information), HRTEM, HAADF‐STEM, EDX mappings (Figure S20, Supporting Information), and Ni 2p XPS (Figure S21, Supporting Information) confirm that the carbon support, Ni composition, and Ni^δ+^ valence state (0<δ<2) remain unchanged after 100 h of electrolysis reaction, preserving its structural integrity and catalytic performance. These findings underscore the robustness and efficiency of the Ni‐N_2_‐CHF electrode for ECO_2_RR.

**Figure 4:**
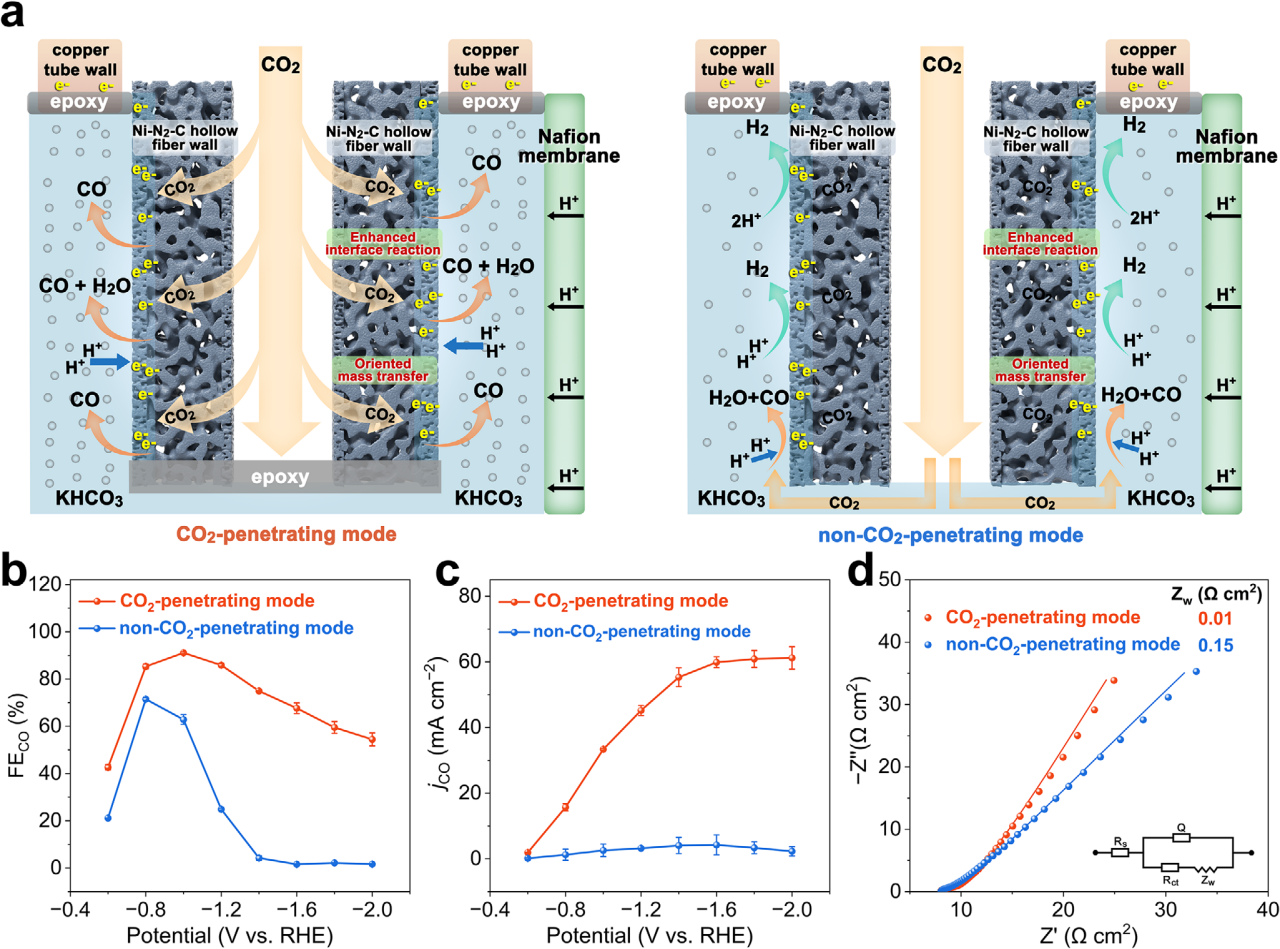
a) Schematic illustration of ECO_2_RR processes over Ni‐N_2_‐CHF electrode with CO_2_‐penetrating mode and non‐CO_2_‐penetrating mode, and their b) CO faradaic efficiencies, c) CO partial current densities, and d) EIS Nyquist plots.

The Ni‐N_2_‐CHF electrode distinguishes itself from other reported self‐supporting electrodes due to its unique gas‐penetration mechanism. With the end sealed, the confined environment of the Ni‐N_2_‐CHF forces gaseous CO_2_ molecules to interact with the Ni‐N_2_ active sites as they penetrate through its porous walls (**Figure** **4**a). This enforced interaction enhances the three‐phase interfacial reaction, optimizing the kinetics of CO production. However, in the absence of CO_2_ penetration (non‐CO_2_‐penetrating mode), this effect is lost (Figure 4a). With the end open, the reactants are predominantly dissolved CO_2_ molecules and reduced at the solid‐liquid two‐phase interface, which will significantly decrease the ECO_2_RR efficiency due to the extremely low CO_2_ solubility and limited mass transfer. Consequently, Ni‐N_2_‐CHF in the CO_2_‐penetrating mode consistently exhibits a higher FE_CO_ across the entire applied potential range compared to the non‐CO_2_‐penetrating mode (Figure 4b). Moreover, the *j*_CO_ in the CO_2_‐penetrating mode increases gradually with more negative applied potentials, with a peak of 61.2 mA cm^−2^ at −2.0 V vs. RHE, In stark contrast, the non‐CO_2_‐penetrating mode delivers extremely low *j*_CO_ values, with a maximum value of just 3.92 mA cm^−2^ at −1.0 V vs. RHE (Figure 4c). Interestingly, the FE_CO_ and *j*_CO_ values for the non‐CO_2_‐penetrating mode are comparable to those of the CO_2_‐penetrating mode at −0.6 and −0.8 V vs. RHE. However, as the potential becomes more negative, these values diverge gradually, with the *j*_CO_ in the CO_2_‐penetrating mode being approximately 27 times higher than that of the non‐CO_2_‐penetrating mode at −2.0 V vs. RHE. These results suggest that at lower potentials, the available CO_2_ molecules at the electrode surface are sufficient to match the limited electron supply, resulting in comparable FE_CO_ and *j*_CO_ values in both modes. However, as the potential negatively shifts and the electron supply increases, the localized CO_2_ availability becomes inadequate in the non‐penetrating mode and HER becomes dominant, resulting in diminished selectivity as well as activity for CO production. On the contrary, the Ni‐N_2_‐CHF electrode in the CO_2_‐penetrating mode can ensure sufficient localized CO_2_ supply for reaction even at higher negative potentials, resulting in superior ECO_2_RR performance.

Furthermore, EIS measurements elucidate the kinetics of ECO_2_RR (Figure 4d). The R_ct_ of Ni‐N_2_‐CHF in the CO_2_‐penetrating mode (1.5 Ω cm^2^) is lower than that in the non‐CO_2_‐penetrating mode (5.6 Ω cm^2^), indicating enhanced charge transfer capability in the CO_2_‐penetrating mode. In addition, the Warburg resistance (Z_w_) for the CO_2_‐penetrating mode (0.01 Ω cm^2^) is remarkably lower than that of the non‐penetrating mode (0.15 Ω cm^2^), highlighting improved mass transport. This enhancement arises from the vigorous bubble perturbation in the solution (Figure S11c, Supporting Information), which promotes mass transport from the bulk electrolyte to the electrode surface. Conversely, the non‐CO_2_‐penetrating mode is dominated by diffusion‐controlled mass transport. These enhanced charge transfer and mass transport characteristics in the CO_2_‐penetrating mode are ascribed to the unique three‐dimensional hierarchical pore structure of the Ni‐N_2_‐CHF electrode, resulting in significantly higher *j*_CO_ values compared to the non‐CO_2_‐penetrating mode (Figure 4c).

**Figure 5:**
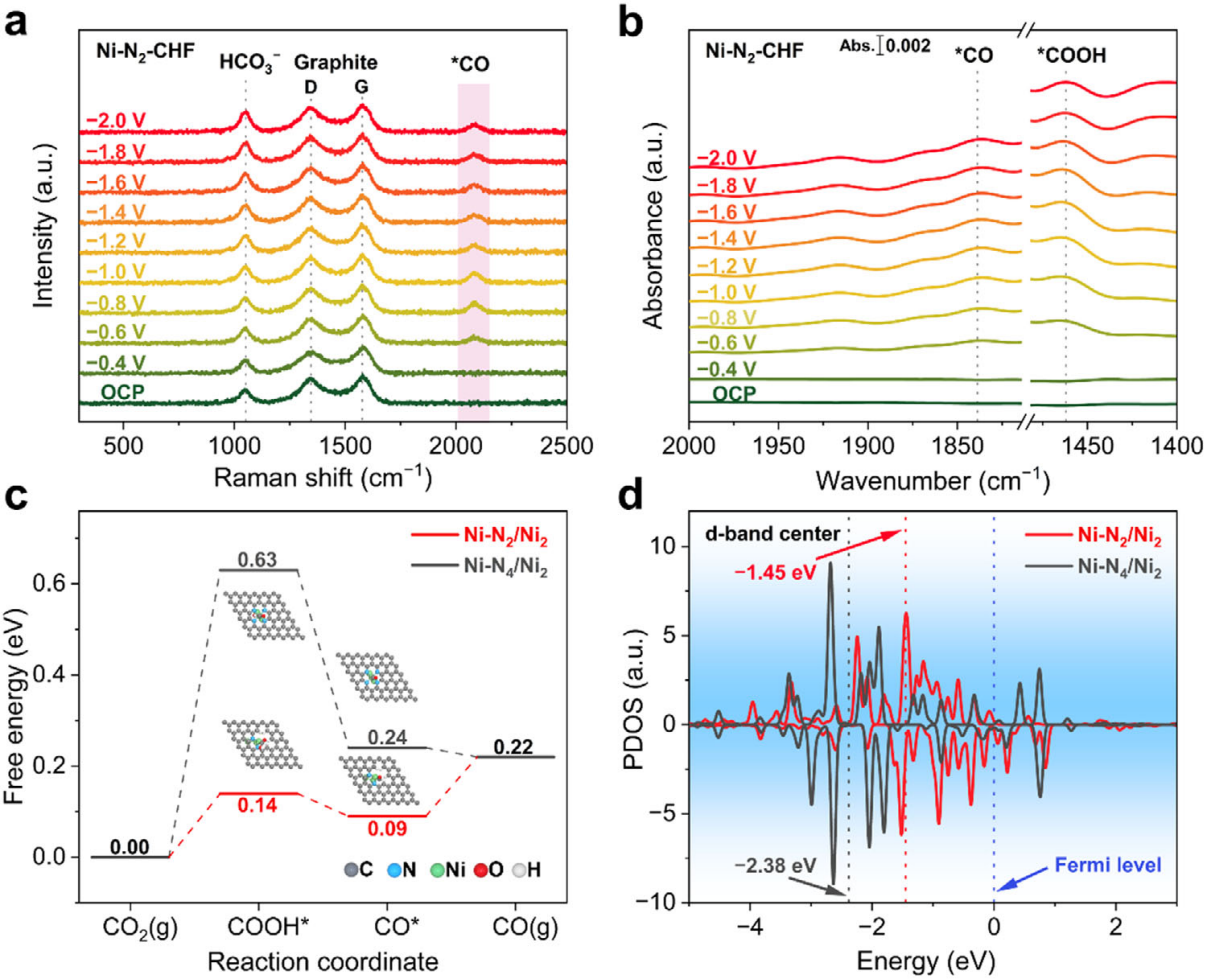
a) In situ Raman spectra and b) ATR‐SEIRAS spectra of Ni‐N_2_‐CHF under ECO_2_RR with increasing potentials. c) The calculated free‐energy diagram for CO_2_ conversion to CO over Ni‐N_2_/Ni_2_ and Ni‐N_4_/Ni_2_. d) PDOS of Ni 3d orbitals on Ni‐N_2_/Ni_2_ and Ni‐N_4_/Ni_2_.

Attenuated total reflection surface‐enhanced infrared absorption spectroscopy (ATR‐SEIRAS) and in situ Raman spectroscopy were used to shed light on the surface reaction intermediates and uncover the basic mechanisms underlying the high ECO_2_RR performance on Ni‐N_2_‐CHF. Sequentially applied potentials ranging from open circuit potential (OCP) to −2.0 V vs. RHE were used to acquire in situ Raman spectra (**Figure** **5**a; Figure S23, Supporting Information). For the Ni‐N_2_‐CHF, Ni‐N_4_‐CHF, and NCHF electrodes, the D and G peaks at 1345 cm^−1^ and 1576 cm^−1^, respectively, remain essentially stable under in situ conditions, indicating the structural stability of both sp^3^ and sp^2^ hybridized carbon species. In situ Raman spectra were also used to detect intermediates associated with the ECO_2_RR on the hollow fiber electrodes. The peak at 2083 cm^−1^ is assigned to the stretching vibration of the *CO,^[^ ^44^, ^45^ ^]^ which is a key intermediate in CO_2_ conversion to CO. For the Ni‐N_2_‐CHF electrode, the *CO signal appears at −0.6 V vs. RHE, while it emerges at −1.0 V vs. RHE and −1.6 V vs. RHE for the Ni‐N_4_‐CHF and NCHF electrodes, respectively. This observation illustrates that Ni‐N_2_‐CHF has a lower onset potential for ECO_2_RR compared to other electrodes. Additionally, the peaks at 1462 cm^−1^ and 1839 cm^−1^ in the ATR‐SEIRAS spectra (Figure 5b; Figure S25, Supporting Information) correspond to the key intermediates *COOH and *CO,^[^ ^45^, ^46^ ^]^ respectively, further confirming the production of CO on the Ni‐N_2_‐CHF electrode.

DFT calculations were conducted to further investigate the origin of the high efficiency of ECO_2_RR on the Ni‐N_2_‐CHF electrode. Based on experimental characterizations, model structures representing Ni‐N_2_‐CHF and Ni‐N_4_‐CHF electrodes were constructed as Ni‐N_2_/Ni_2_ and Ni‐N_4_/Ni_2_, respectively, for computational analysis (Figure S26, Supporting Information). The constructed Ni‐N_2_/Ni_2_ and Ni‐N_4_/Ni_2_ models were further undergone Ni K‐edge XANES simulations. The fitting theoretical XANES spectra well match the experimental XANES spectra of Ni‐N_2_‐CHF and Ni‐N_4_‐CHF electrodes (Figure S27, Supporting Information), indicating the rationality of the constructed Ni‐N_2_/Ni_2_ and Ni‐N_4_/Ni_2_ models.

The possible reaction pathways for CO_2_ electroreduction to CO were first investigated, encompassing the following four elementary steps (* = catalytic site):

(1) $$CO_{2}+*\;CO_{2}*$$

(2) $$CO_{2}*+H^{+}+e^{\_}\;\mathrm{COO}H^{*}$$

(3) $$\mathrm{COO}H^{*}+H^{+}+e^{\_}\;CO^{*}+H_{2}O$$

(4) $$CO^{*}\to \;\mathrm{CO}+*$$

As revealed by the free energy calculations for these steps on two electrodes (Figure 5c), the COOH* formation step is the most energy‐intensive step, requiring 0.14 and 0.63 eV for Ni‐N_2_‐CHF and Ni‐N_4_‐CHF electrodes, respectively. This confirms COOH* formation as the rate‐determining step, which accords with Tafel analysis results. The unsaturated Ni‐N_2_ coordination structure in Ni‐N_2_‐CHF effectively reduces the energy barrier for COOH* formation, improving catalytic activity and selectivity. Moreover, the Ni‐N_2_ structure facilitates the water activation, generating hydrogen species readily reacting with CO_2_ to form COOH* and subsequently CO*, as evidenced by the reduced energy barrier for hydrogen species formation (Figures S28, S29, Supporting Information). The COOH* to CO* conversion is an exothermic process on both sites. Although CO* desoprtion on the Ni‐N_2_ site is an endothermic process with an energy barrier of 0.13 eV, the overall CO_2_ to CO conversion reaction is still more favorable on Ni‐N_2_ site than on the Ni‐N_4_ site, consistent with the experimental results.

Charge difference density analysis for COOH* on Ni‐N_2_/Ni_2_ (Figure S30, Supporting Information) shows greater charge accumulation compared to Ni‐N_4_/Ni_2_, which reveals better adsorption of COOH* on Ni‐N_2_/Ni_2_. Moreover, the projected density of states (PDOS) of the Ni 3d orbitals of Ni‐N_2_/Ni_2_ demonstrates a significant positive shift in the d‐band center relative to Ni‐N_4_/Ni_2_ (Figure 5d). This shift aligns the energy level of Ni 3d orbital electrons closer to the Fermi energy level, enhancing their reactivity.^[^ ^47^ ^]^ Bader charge analysis further reveals that the Ni valence state in Ni‐N_2_/Ni_2_ (+0.51) is lower than that in Ni‐N_4_/Ni_2_ (+0.81) (Table S7, Supporting Information), corroborating XANES results. These findings indicate that the active Ni‐N_2_ sites in Ni‐N_2_‐CHF exhibit higher electron density, which promotes the adsorption of key intermediates and markedly reduces the energy barrier for CO formation.

## 3 Conclusion

In summary, we successfully developed a carbon hollow fiber with an unsaturated Ni‐N_2_ coordination structure (Ni‐N_2_‐CHF). This material functions as a self‐supporting gas penetration electrode exhibiting superior electrocatalytic performance for CO_2_ reduction. It achieves a high CO Faradaic efficiency (exceeding 90%) and an excellent long‐term stability of over 100 h, surpassing previously reported carbon supporting electrodes with Ni‐N‐C sites. Comparative experiments reveal that the unique hollow fiber penetration electrode configuration significantly enhances charge transfer and mass transport, leading to favorable ECO_2_RR kinetics and thus high CO formation activity. In situ experimental analyses combined with DFT calculations further show that the Ni‐N_2_ structure in Ni‐N_2_‐CHF lowers the energy barriers for COOH* intermediate formation, thereby facilitating CO_2_ electroreduction. Our work highlights a promising strategy for developing self‐supporting carbon electrodes with tailored active sites to achieve superior CO_2_ electroreduction performance.

## Conflict of Interest

The authors declare no conflict of interest.

## Supporting information



Supporting Information

## Data Availability

The data supporting the findings of this study are available from the corresponding author upon reasonable request.
