# Supplementary material for: Carbon Hollow Fiber Penetration Electrode with Unsaturated Ni‐N2 Coordination for Enhanced CO2 Electroreduction
Source: Adv Sci (Weinh). 2025 May 20;12(29):e02947. doi: 10.1002/advs.202502947 (PMC12362814; doi:10.1002/advs.202502947)
Supplement: Supplementary file 1 — Supporting Information [file ADVS-12-e02947-s001.pdf]

ADVANCED SCIENCE
Open Access

Supporting Information

for *Adv. Sci.*, DOI 10.1002/advs.202502947

Carbon Hollow Fiber Penetration Electrode with Unsaturated Ni-N_2 Coordination for Enhanced CO_2 Electroreduction

Xiaotong Wang, Yiheng Wei, Yanfang Song, Jianing Mao, Xiaohu Liu, Shoujie Li, Guihua Li, Huanyi Zhu, Jiayu Xia, Cheng Luo, Aohui Chen, Xiao Dong, Wei Wei* and Wei Chen**

Supporting Information

Carbon Hollow Fiber Penetration Electrode with Unsaturated Ni-N_2 Coordination for Enhanced CO_2 Electroreduction

Xiaotong Wang[1,2,3,+], *Yiheng Wei*[1,2,3,+], *Yanfang Song*[1,2,3*], *Jianing Mao*[1,4], *Xiaohu Liu*[1,3,5], *Shoujie Li*[1,2,3], *Guihua Li*[1,2,3], *Huanyi Zhu*[1,2,3], *Jiayu Xia*[1,2,3], *Cheng Luo*[1,2,3], *Aohui Chen*[1,3,5], *Xiao Dong*[1,2,3], *Wei Wei*[1,2,3,5*] and *Wei Chen*[1,2,3*]

[1]Low-Carbon Conversion Science and Engineering Center, Shanghai Advanced Research Institute, Chinese Academy of Sciences, Shanghai 201210, PR China

[2]University of Chinese Academy of Sciences, Beijing 100049, PR China

[3]State Key Laboratory of Low Carbon Catalysis and Carbon Dioxide Utilization, Shanghai Advanced Research Institute, Chinese Academy of Sciences, Shanghai 201210, PR China

[4]Shanghai Institute of Applied Physics, Chinese Academy of Sciences, Shanghai, 201204, PR China

[5]School of Physical Science and Technology, ShanghaiTech University, Shanghai 201203, PR China

[+]These authors contributed equally to this work.

*Corresponding author. E-mail: songyf@sari.ac.cn, weiwei@sari.ac.cn, chenw@sari.ac.cn

Table of Contents.. 4

Materials and methods 4

Chemicals and Materials 4

Preparations 4

Characterizations 5

Electrochemical measurements 6

Product quantification 7

Computational section 9

Statistical Analysis 9

Supporting Figures 11

Figure S1 11

Figure S2 12

Figure S3 13

Figure S4 14

Figure S5 15

Figure S6 16

Figure S7 17

Figure S8 18

Figure S9 19

Figure S10 20

Figure S11 21

Figure S12 22

Figure S13 23

Figure S14 24

Figure S15 25

Figure S16 26

Figure S17 27

Figure S18 28

Figure S19 29

Figure S20 30

Figure S21 31

Figure S22 32

Figure S23.. 33
Figure S24.. 34
Figure S25. 35
Figure S26.. 36
Figure S27.. 37
Figure S28.. 38
Figure S29.. 39
Figure S30.. 40
Supporting Tables.. 41
Table S1.... 41
Table S2.... 42
Table S3.... 43
Table S4.... 44
Table S5..... 45
Table S6.... 46
Table S7.... 47
References.. 48

Materials and methods

Chemicals and Materials

PAN (Polyacrylonitrile), N-methyl-2-pyrrolidone (NMP), and nickel nitrate hexahydrate ($Ni(NO_3)_2 \cdot 6H_2O$) were purchased from Sinopharm Chemical Reagent Co.,Ltd. Nafion 117 membrane was purchased from DuPont. $KHCO_3$, ethanol, and melamine were purchased from Shanghai Titan Scientific Co., Ltd. Dimethyl sulfoxide was purchased from Sigma-Aldrich. All chemicals were used without further purification. Electrolyte solutions were prepared using 18.2 MΩ H_2O (EU-40UVF Water Purification System).

Preparations

Preparation of carbon hollow fiber (CHF)

CHF was prepared by a phase-inversion and carbonization process (Figure S1). PAN and NMP were used as the carbon precursor and the solvent, respectively. Briefly, PAN and NMP were mixed in a weight ratio of 1:7.5 using ball-mailing (500 rpm) for 20 h to form a homogeneous slurry. Subsequently, the acquired slurry was vacuumed to degas after cooling to room temperature. Next, the casting liquid was excluded through the spinning machine and shaped in the tap water bath by phase-inversion. After spinning, the molded tubes were immersed in a water bath for 24 h for removal of NMP solvent, followed by stretching and drying for 24h to obtain a green body. Then, the green body was cut into appropriate lengths and heated in a tubular furnace under an argon atmosphere (50 sccm) at 350 ^{o}C for 2 h, followed by 900 ^{o}C for 2 h. The heating process involved a ramping rate of 1°C min^{-1} below 350°C and 5°C min^{-1} between 350 °C and 900 °C to obtain the CHF.

Preparation of Ni-N_2-CHF, Ni-N_4-CHF, and nitrogen-doped carbon hollow fiber (NCHF) electrodes

Ni-N_2-CHF was prepared from CHF through Ni^{2+} adsorption, melamine coating and calcination, as outlined in Figure S1. Typically, CHF was immersed in a 10 mL reaction tube containing a 2 M of nickel nitrate ($Ni(NO_3)_2$) solution under vacuum for 4 h to adsorb Ni^{2+}. The fiber was then dried in an oven at 80°C and washed with deionized water to remove excess Ni^{2+}. After drying in a vacuum oven at 60 ^{o}C, the fiber was coated with melamine by dipping in a 16

mg mL^{-1} of melamine/ethanol dispersion obtained via ultrasonic processing for 30 minutes. The melamine-coated fiber was further dried in an oven at 70 ^{o}C and then calcined at 800 ^{o}C for 1 h under an argon atmosphere (60 sccm) with a ramp rate of 5 ^{o}C min^{-1} to acquire Ni-N_2-CHF. For comparison, Ni-N_4-CHF and NCHF were also synthesized using similar procedures, with the only difference being the elimination of the melamine coating step for Ni-N_4-CHF and the removal of the Ni^{2+} adsorption step for NCHF.

Characterizations

The cross-section and surface morphology of the hollow fiber were observed by scan electron microscopy (SEM) using a Verios G4 UC with an accelerating voltage of 2.0 kV. High-Resolution Transmission Electron Microscopy (HRTEM) observations were carried out on a JEOL-F200 at 200 kV. The High-angle annular dark-field scanning TEM (HAADF-STEM) combined with EDX measurements were performed on a Spectra 300S/TEM, FEI, at 200kV. X-ray diffraction (XRD) measurements were performed on a Rigaku Ultima 4 X-ray diffractometer using Cu Kα radiation (λ = 1.54056 Å) under 40 kV, 40 mA, and analyzed in the 2θ range of 5° to 90° with a scanning speed of 4° min^{-1}. X-ray photoelectron spectroscopy (XPS) was performed on a Quantum 2000 scanning ESCA microprobe instrument using a monochromatic Al Kα source (1486.6 eV). The binding energies in all XPS spectra were calibrated against the C 1s peak (284.8 eV). Raman spectra were obtained on a HORIBA LabRAM HR Evolution Raman spectrometer using a 532 nm laser excitation. Inductively coupled plasma (ICP) measurements were performed on an optical emission spectrometer (AGILENT ICP-OES 730). The standard solution containing the element of Ni was selected during analysis. X-ray adsorption fine structure spectroscopy (XAFS) measurements at the Ni K-edge were performed in transmission mode in the BL14W1 station at the Shanghai Synchrotron Radiation Facility (SSRF, 3.5 GeV, 220 mA maximum, Si(111) double crystals). The XAFS raw data were background-subtracted and normalized by the ATHENA program. Curve-fitting analysis of EXAFS χ(k) data (including different coordination shells, in the *R*-space of 1.0-3.0 Å) and Fourier transforms (in the *K*-space of 3.0-12.0 $Å^{-1}$) were carried out by using the ARTEMIS program. The Ni K-edge XANES simulation was performed based on FDMNES code with multiple scattering modes (Green) using the muffin-tin potential. The calculated radius was 6.5 Å with a self-consistent calculated radius of 6 Å. Theoretical scattering amplitudes, phase shifts, and the photoelectron mean free path for all paths were calculated using the Hanning window (dk = 1.0 $Å^{-1}$) in the IFEFFIT package. Electron

paramagnetic resonance (EPR) spectra were recorded on a Brucker A300 spectrophotometer equipped with an Oxford Instruments liquid helium cryostat, using 1-20 mW microwave power and 100 kHz field modulation with the amplitude set to 1 G. The g-values for each EPR spectrum were extracted from simulations performed using EasySpin (v5.2.23).

Electrochemical measurements

Each hollow fiber was stuck into a copper tube with conductive silver adhesive to make electrical contact. The opposing end of the hollow fiber tube and the joints between the fiber and the copper tube were sealed and covered with gas-tight and non-conductive epoxy. After drying for 12 h at room temperature, a self-supporting hollow fiber working electrode was obtained with an exposed length of 1.0 cm. The geometric area was calculated using the equation $S = \pi D_{out}L$, where S was the electrode area, D_{out} was the outer diameter of the hollow fiber, and L was the length of the hollow fiber.

Electrochemical studies of the prepared hollow fiber were evaluated at ambient temperature and pressure on the Biologic VMP3 potentiostat by using a gas-tight electrolysis cell assembled with a KCl-saturated Ag/AgCl reference electrode and a platinum mesh ($3 \times 3 cm^2$) counter electrode. The cathode and anode compartments were separated by a Nafion 117 membrane. Both catholyte and anolyte employed 0.5 M $KHCO_3$ aqueous solution. CO_2 was continuously delivered through each hollow fiber in the cathodic compartment at a constant rate of 10 sccm. Linear Sweep voltammetry (LSV) measurements were performed with a scan rate of 10 mV s^{-1}. The CO_2 reduction was conducted under the potentiostatic mode for 20 min at each potential. All of the applied potentials were recorded against a KCl-saturated Ag/AgCl reference electrode and converted to values versus reversible hydrogen electrode (RHE) with iR loss correction using E (*vs.* RHE) = E (*vs.* Ag/AgCl) + 0.197 V + 0.0591 V × pH - iR. All potentials refer to RHE unless otherwise noted. The electrochemical active surface area (ECSA) of the electrode was measured by double-layer capacitance (C_{dl}). C_{dl} was determined by cyclic voltammetry (CV) at potentials ranging from −0.005 to 0.095 V (*vs.* RHE), with corresponding scan rates of 50, 40, 30, 20 and 10 mV·s^{-1}. The slope of the plot current density ((j_a-j_c)/2) against the scan rate yielded the value of C_{dl}. The electrochemical impedance spectroscopy (EIS) was performed at open circuit potential (OCP). The frequency limits were typically set from 0.1 Hz to 1000 kHz with a voltage amplitude of 5 mV. Before the experiments, the electrolysis cell

was purged with CO_2 for 30 min, followed by continuous delivery of CO_2 through the interior of the hollow fiber to the cathodic compartment at a constant rate of 10 mL min^{-1}.

Product quantification

Gas-phase products generated from the cathodic compartment during the electrochemical reactions were directly vented into the gas chromatograph (GC-2014, Shimadzu) equipped with a Molecular sieve-13X 60/80 column and a Plot-Q80/100 column and analyzed online. H_2 was analyzed using a thermal conductivity detector (TCD), and CO was analyzed using a flame ionization detector (FID). A GC run was initiated every 10 min. High-purity nitrogen (99.999%) was used as the carrier gas. Only H_2 and CO were found in the gas-phase products for all CO_2 electrolysis tests. The faradaic efficiencies of the gas products were calculated from the concentration (ppm) detected by the GC as follows:

$$\boldsymbol{FE = \frac{C_{product} \times 10^{-6} \times v \times 10^{-3} \times t \times \alpha \times 96485}{22.4 \times Q} \times 100\%}$$

Where C_{product} (ppm) is the concentration of the gas-phase products, v (sccm) is the flow rate of the CO_2, t (s) is the reaction time, α is the number of transferred electrons for producing CO or H_2 and Q (A·s) is the total quantity of electric charge.

After each potentiostatic electrolysis experiment for 20 min, liquid-phase products from the cathodic compartment were analyzed and quantified by ^{1}H NMR spectra using a 600 MHz NMR spectrometer (Bruker). Before measurement, 0.5 mL 0.5 M $KHCO_3$ catholyte solution after CO_2 electrolysis was mixed with 0.1 mL of 5 mM DMSO (HPLC grade, Sigma-Aldrich) in D_2O stock solution. As confirmed by ^{1}H NMR spectra (Figure S12), no liquid product formed after CO_2 electrolysis over the prepared electrodes.

In situ attenuated total reflection surface-enhanced infrared absorption spectroscopy (ATR-SEIRAS) measurements

The electrochemical in situ ATR-SEIRAS measurements were conducted using an INVENIO R FTIR spectrometer (Bruker) equipped with a mercury–cadmium–telluride (MCT)

detector. Gold films were chemically deposited on a face-angled Si crystal, serving as the conductive substrate for the surface-enhanced effect. The catalyst ink was drop-casted onto the Au-deposited Si crystal, preparing the working electrode for a custom-made spectroelectrochemical cell fixed on the ATR accessory (Figure S24). A saturated Hg/HgO electrode served as the reference electrode, and a Pt foil was used as the counter electrode.

During spectrum collection, the optical path was continuously purged with nitrogen gas to minimize interference from water and CO_2 in the air. The electrolyte used was 0.5 M $KHCO_3$, and prior to testing, it was purged with pure Ar for 10 minutes. Background measurements were taken at the open circuit potential, after which spectra were collected at different applied potentials. All spectra were presented as absorbance ($-\log(R/R_0)$).

In-situ Raman measurements

A modified electrochemical Raman cell (three-electrode polyvinylidene fluoride cell with a 0.5 mm thick sapphire window) was used in conjunction with a confocal Raman spectrometer (Renishaw inVia) (Figure S22). A 532 nm excitation laser (10% power) at 50 mW was used as the light source, with a grating of 1800, focused on the sample surface through a 50X telephoto objective. During Raman measurements, the distance between the sapphire window and the surface of the working electrode was kept below 0.1 mm, ensuring a very thin solution layer on the electrode surface. This minimized the attenuation of the Raman signal by the solution layer.

Gas Permeation Tests

Gas permeation tests were performed using a customized gas permeability device. The permeabilities of N_2 and CO_2 through the hollow fiber were recorded under different transmembrane pressure drops. The permeation of N_2 and CO_2 was obtained according to the Yasuda-Tsai equation[1,2]. The permeability coefficient K of porous hollow fibers can be expressed as follows:

$$\boldsymbol{K = K_O + B_O P \eta^{-1}}$$

K_o is the Knudsen permeability coefficient, B_o is the geometric factor of the hollow fiber wall, P is the average pressure on both sides of the fiber, and η is the viscosity of N_2 gas. The values of K_o and B_o can be calculated from the slope and intercept of the plot of K to P. The

effective porosity (ε/q^2) can also be estimated from the Knudsen permeability coefficient K_o as follows:

$$\boldsymbol{K_0^2} = \left(\frac{\mathbf{3.2}}{\mathbf{3}}\right)\left(\frac{\boldsymbol{\varepsilon}}{\boldsymbol{q^2}}\right)\frac{\mathbf{16}\boldsymbol{B_0}}{\mathbf{3}}\frac{\mathbf{4}\boldsymbol{RT}}{\boldsymbol{\pi}\mathbf{M}}$$

Where ε is the porosity, q is the tortuosity factor, R is the gas constant, T is the temperature, and M is the molecular weight of the gas.

Computational section

All calculations were based on the density functional theory (DFT) method, as implemented in the Vienna *ab initio* Simulation Package (VASP).[3-5] The frozen-core projector augmented-wave (PAW) potentials were employed for the core and valence electronic interactions.[6] The revised Perdew-Burke-Ernzerhof functional form of the generalized-gradient approximation (GGA) for electron exchange and correlation energies was used throughout.[7,8] The energy cutoff for the plane wave expansion was set to be 450 eV. A vacuum region in the *z*-direction was set as 30 Å in order to avoid interactions between the periodic images. The van der Waals interaction was calculated using the DFT-D3 method.[9] The Monkhorst-Pack method was used for sampling the Brillouin zone with a 2×2×1 mesh. Geometry optimizations for all models were performed with the energy and force convergence criterion of 1×10^{-4} eV and 0.01 eV $Å^{-1}$ per atom, respectively.

The adsorption energies (E_{ads}) were calculated according to the following equation:

$$E_{ads} = E_{total} - E_{substrate} - E_{adsorbate}$$

Where E_{total}, $E_{substrate}$, and $E_{adsorbate}$ are the total energies of substrates with adsorbates, the pristine substrates and adsorbates, respectively.

The reaction Gibbs free energies (ΔG) can be calculated by the following equation:

$$\Delta G = \Delta E + \Delta E_{ZPE} - T\Delta S$$

Where E and E_{ZPE} are the electronic potential and zero-point energy, respectively, T is 298.15 K, and S is the entropy.

Statistical Analysis

The electrochemical performance data (e.g., Faradaic efficiency, Current density) were analyzed using Origin 2025 for statistical processing and visualization. Repeated measurements (at least three independent experiments) were conducted under identical conditions to ensure

reproducibility. The relevant data were presented in the form of mean value ± standard deviation (SD). Error bars in figures represent standard deviations derived from three independent measurements.

Supporting Figures

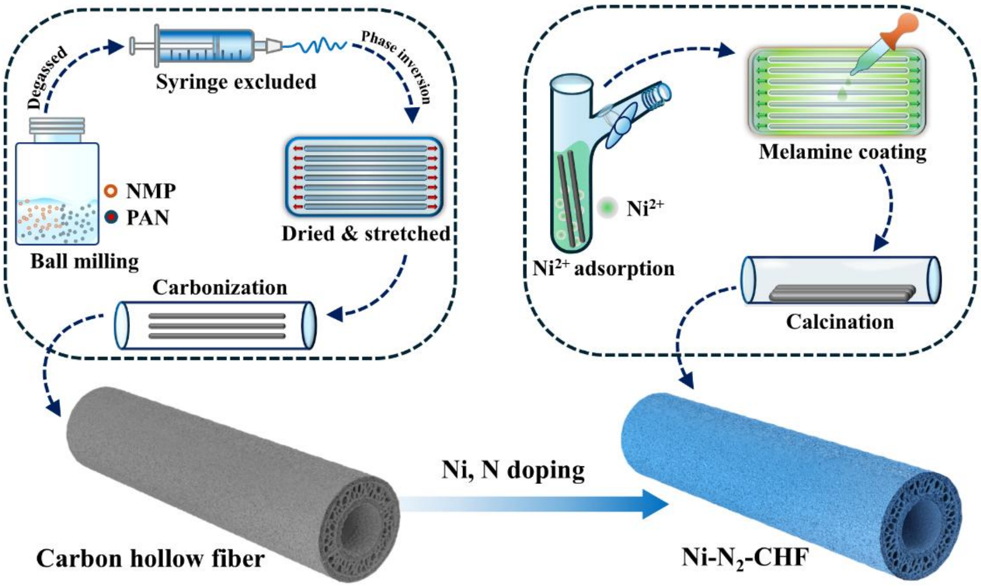

Figure S1. Schematic diagram of the Ni-N_2-CHF preparation process.

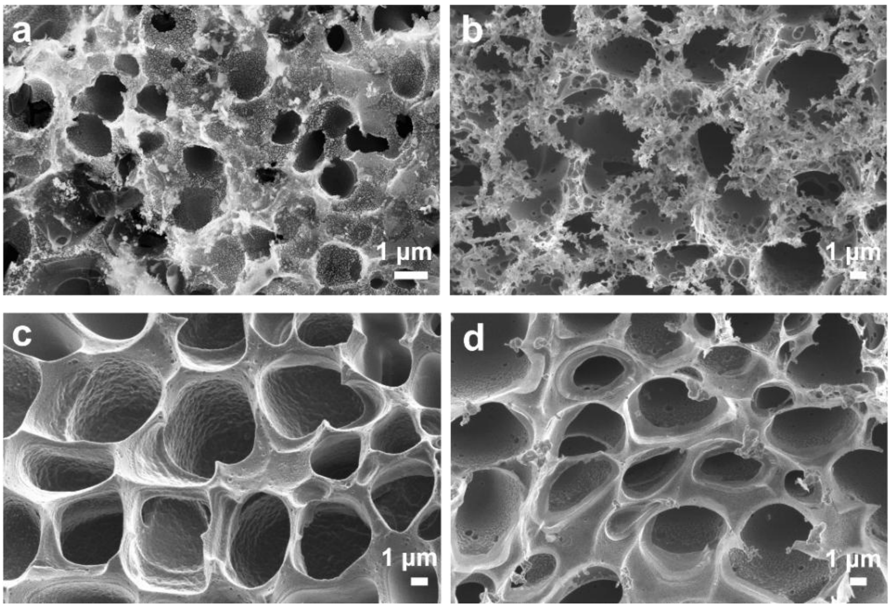

Figure S2. SEM images of the outer surfaces of (a) CHF, (b) NCHF, (c) Ni-N_2-CHF, and (d) Ni-N_4-CHF.

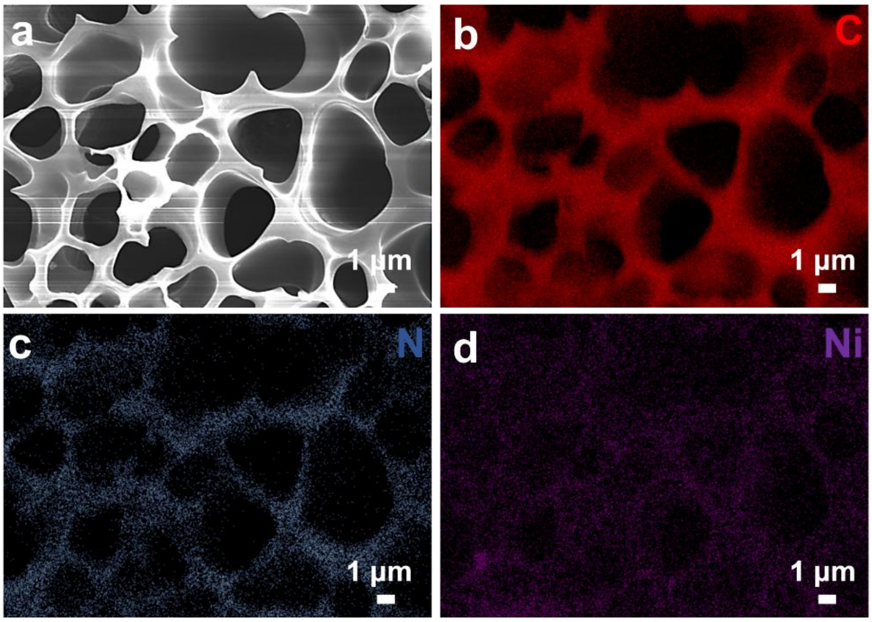

Figure S3. (a) SEM and (b-d) corresponding EDS elemental mapping images for the surface of Ni-N_2-CHF.

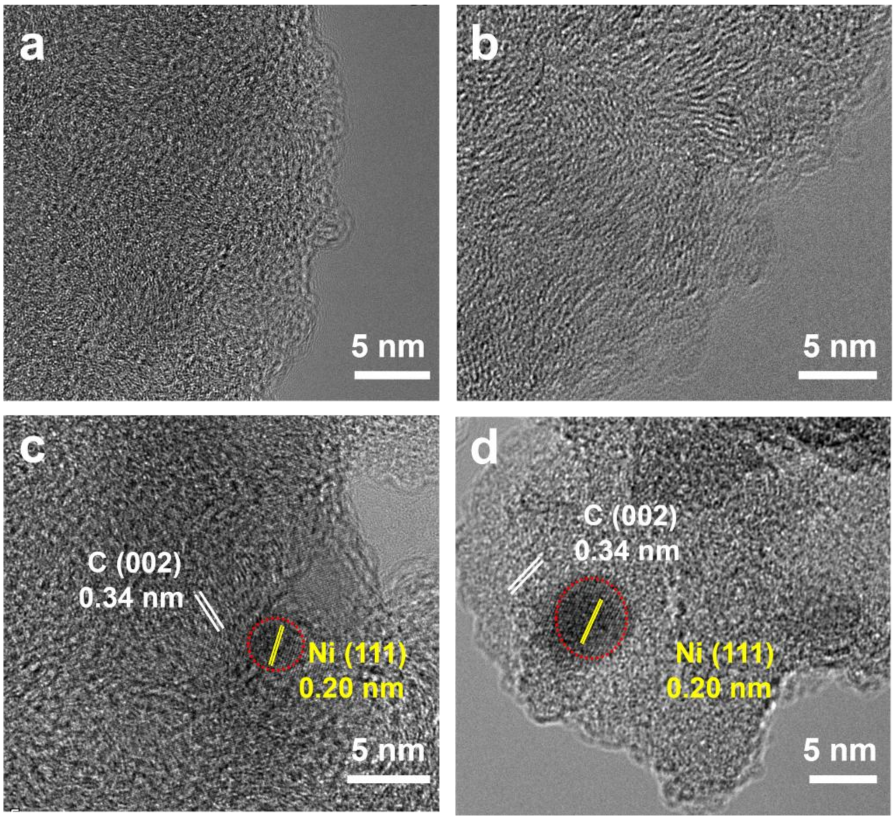

Figure S4. TEM images of (a) CHF, (b) NCHF, (c) Ni-N_2-CHF, and (d) Ni-N_4-CHF.

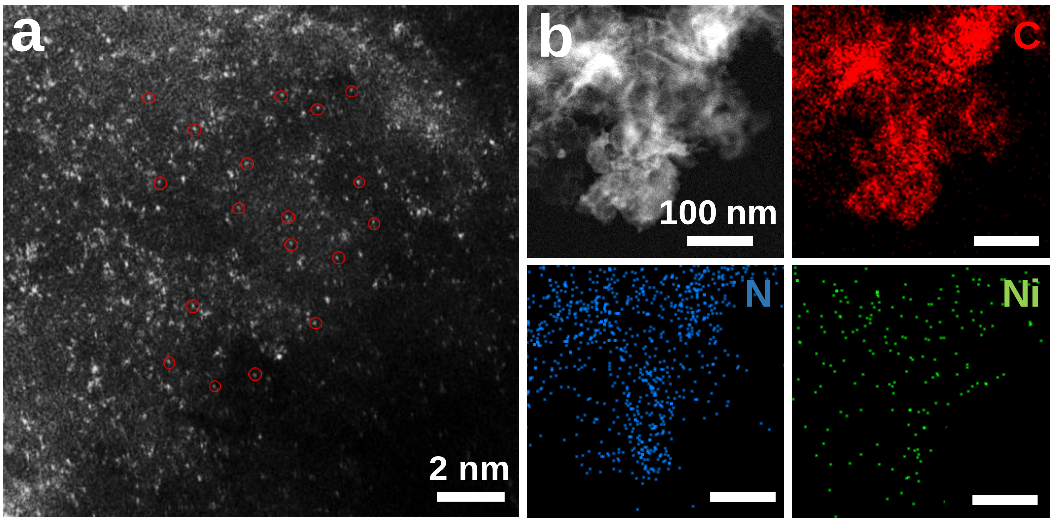

Figure S5. (a) HAADF-STEM image for Ni-N_4-CHF showing Ni single atoms (red circles) and (b) the corresponding element mappings.

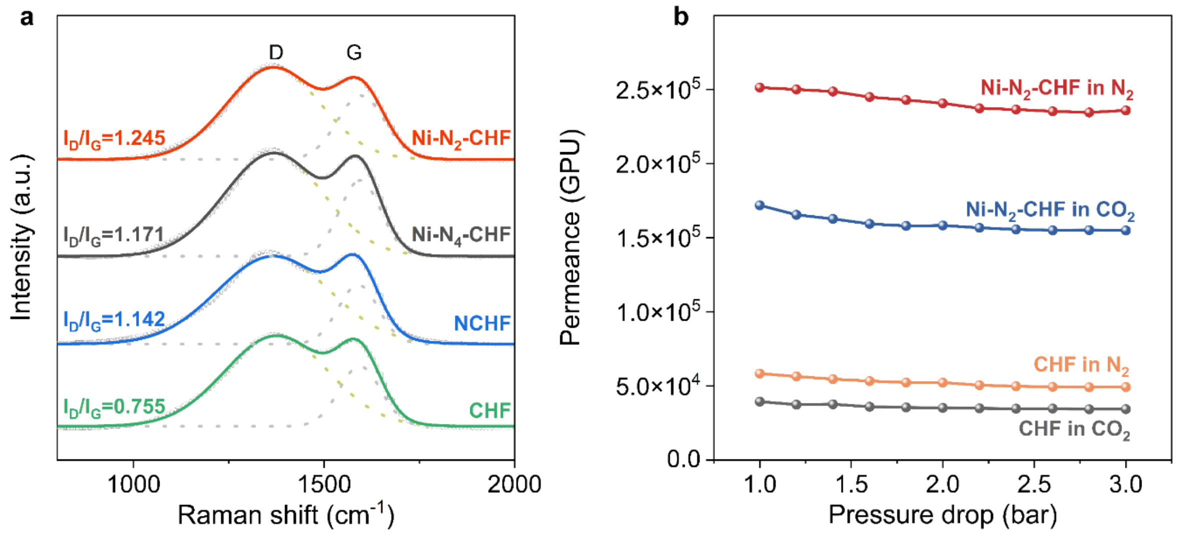

Figure S6. (a) Raman spectra of Ni-N_2-CHF, Ni-N_4-CHF, NCHF, and CHF with the I_D/I_G ratios based on peak fitting. (b) Gas permeances of Ni-N_2-CHF and CHF in N_2 and CO_2 atmosphere at different transmembrane pressure drops.

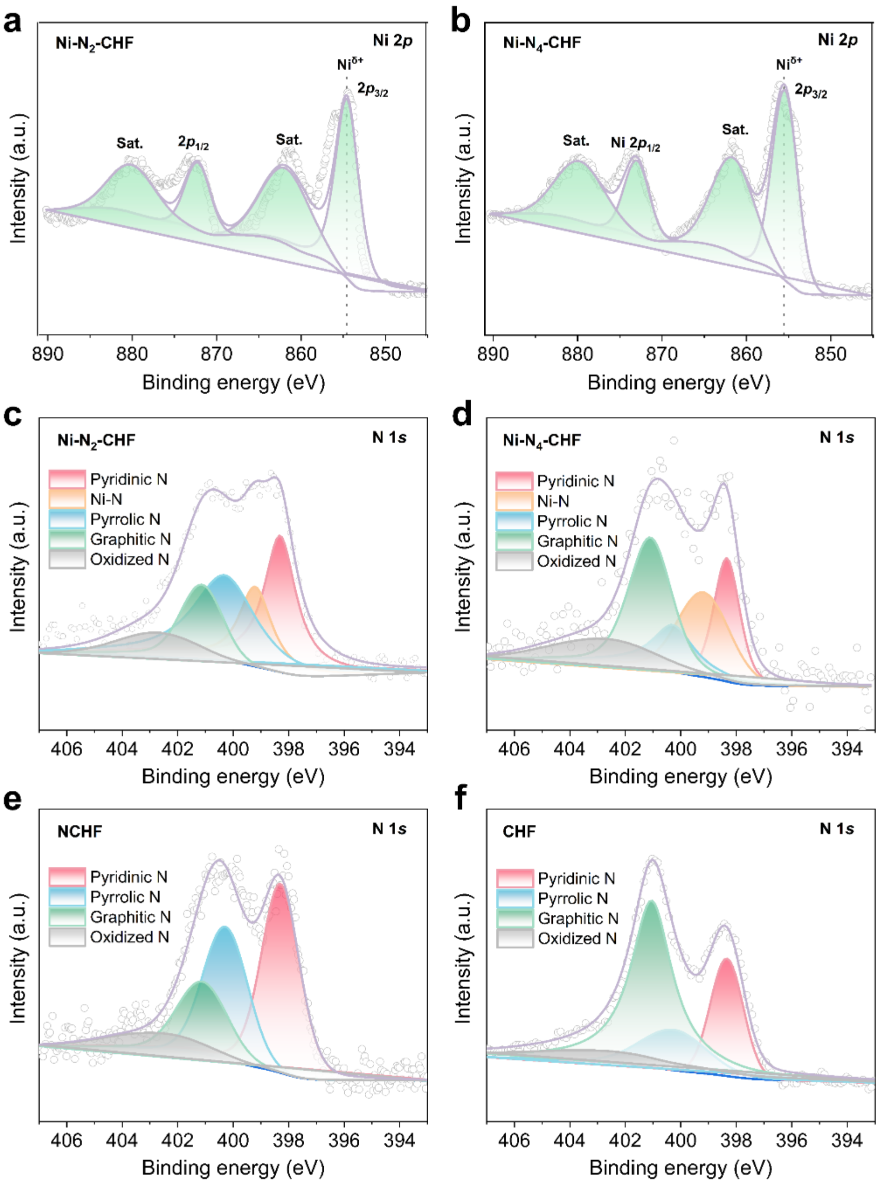

Figure S7. The Ni 2p XPS for (a) Ni-N_2-CHF and (b) Ni-N_4-CHF, and the N 1s XPS for (c) Ni-N_2-CHF, (d) Ni-N_4-CHF, (e) NCHF, and (f) CHF.

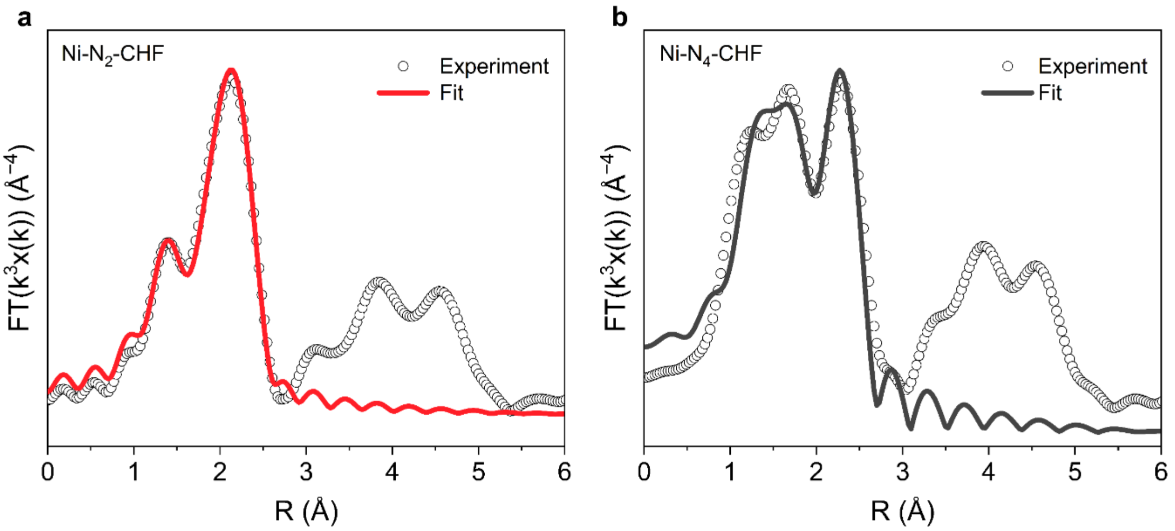

Figure S8. Ni K-edge EXAFS fitting curves of (a) Ni-N_2-CHF and (b) Ni-N_4-CHF.

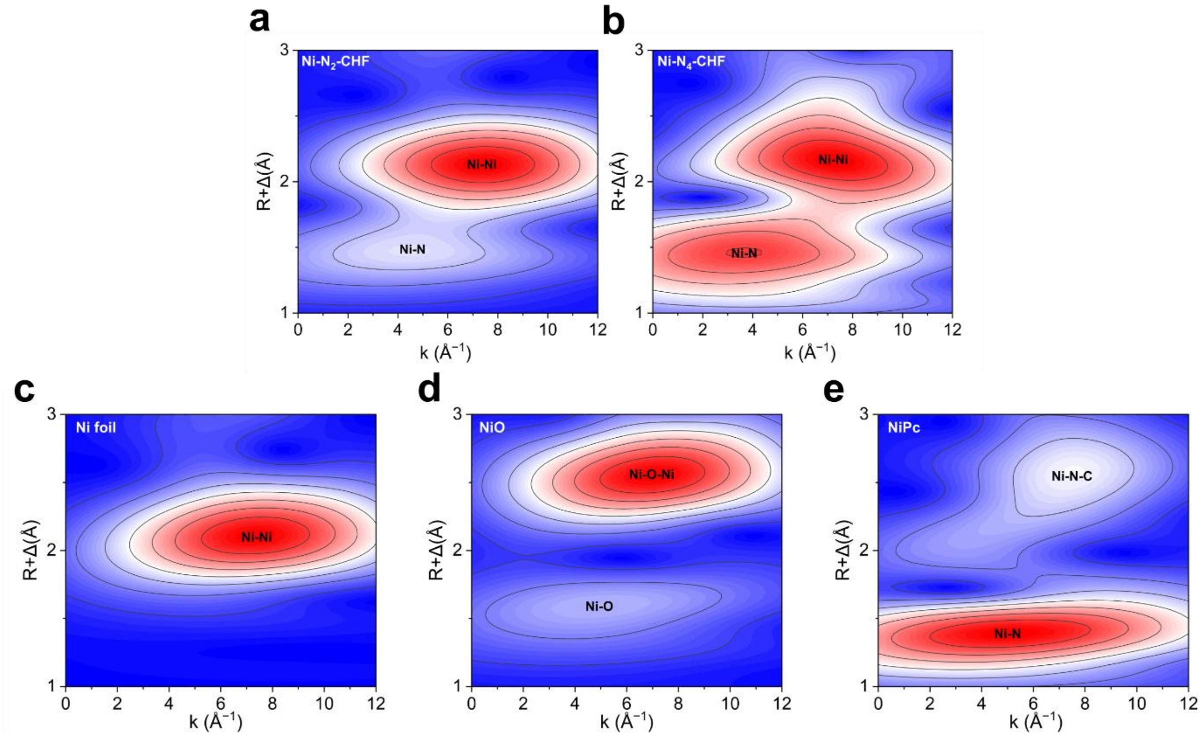

Figure S9. WT-EXAFS contour patterns of (a) $Ni-N_2$-CHF, (b) $Ni-N_4$-CHF, (c) Ni foil, (d) NiO, and (e) NiPc.

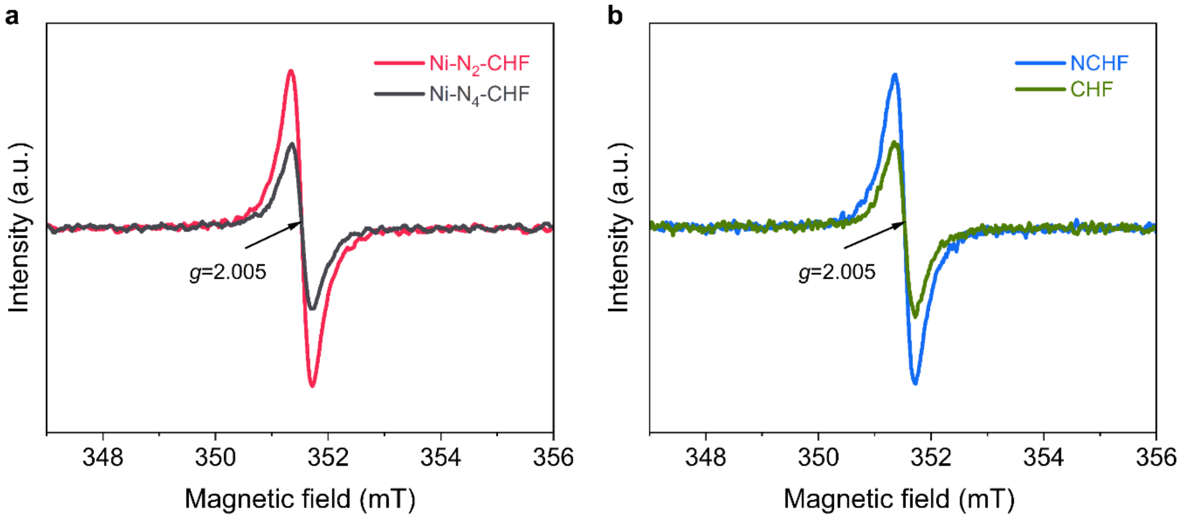

Figure S10. Electron paramagnetic resonance (EPR) spectra of (a) Ni-N_2-CHF and Ni-N_4-CHF, (b) NCHF and CHF.

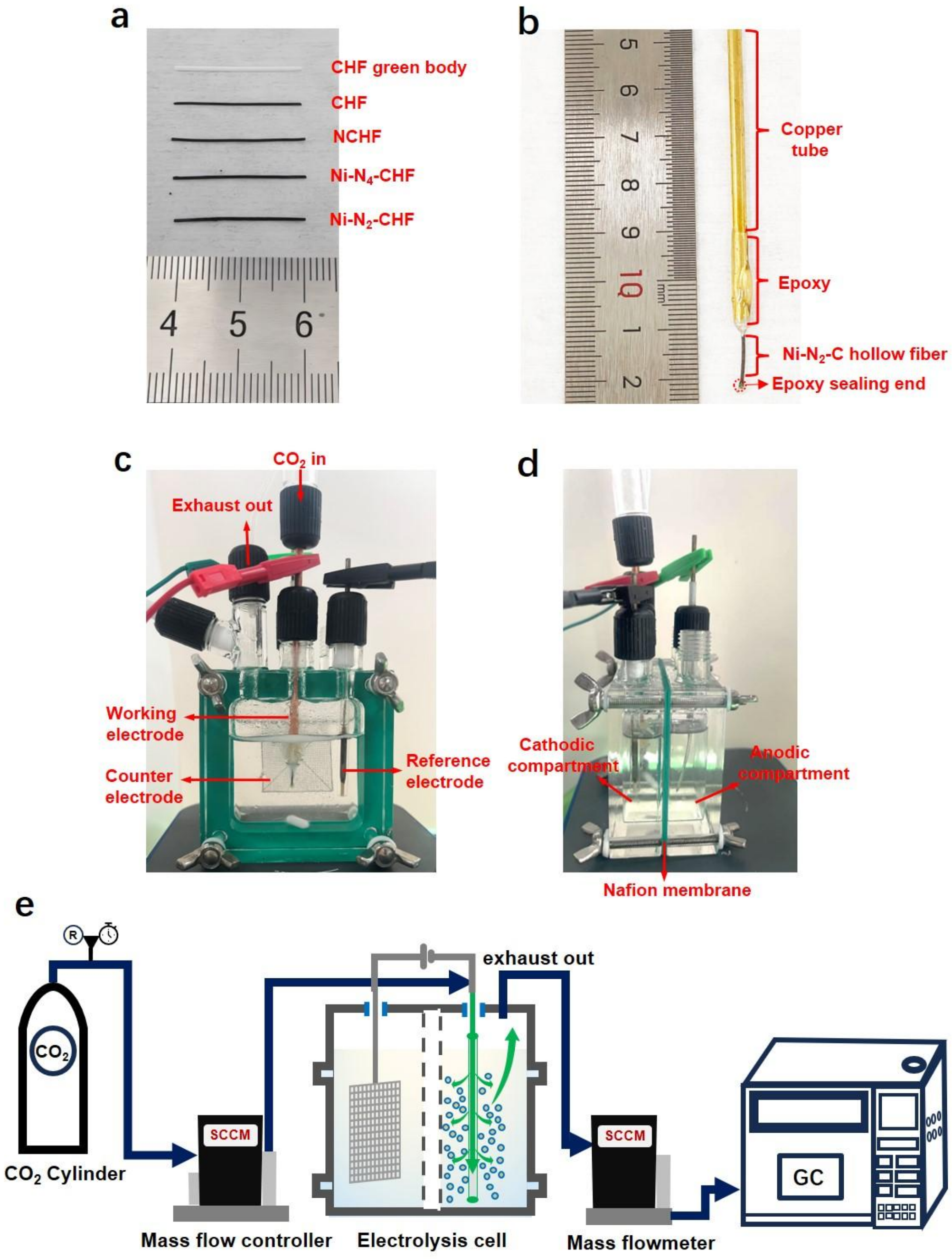

Figure S11. (a) Optical images of CHF green body, CHF, NCHF, Ni-N_4-CHF, and Ni-N_2-CHF. (b) The assembled working electrode of Ni-N_2-CHF. The (c) front view and (d) side view of the gas-tight electrolysis cell during CO_2 electroreduction. (e) the schematic illustration of the electrolysis system for CO_2 electroreduction.

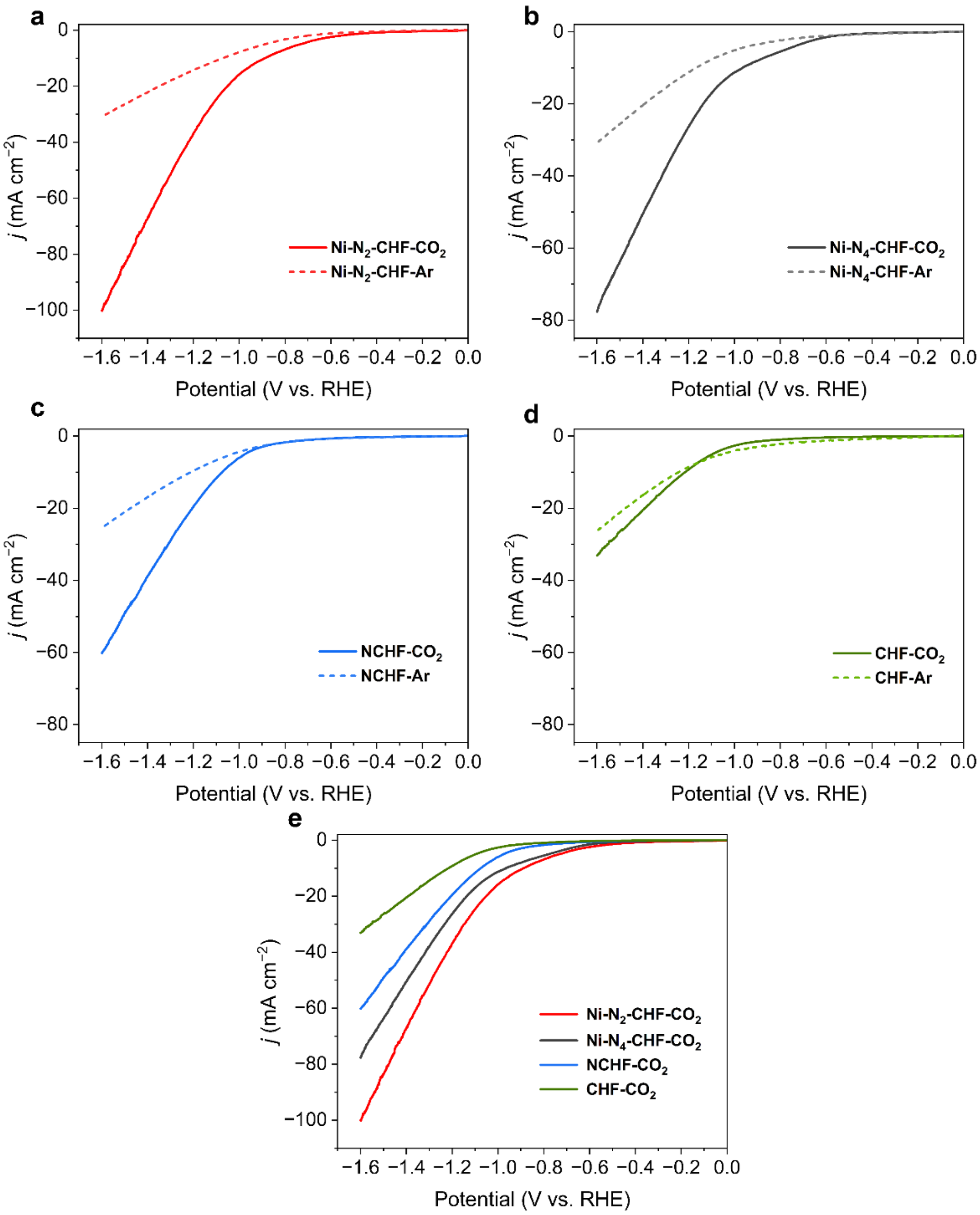

Figure S12. Linear sweep voltammetry (LSV) curves of (a) Ni-N_2-CHF, (b) Ni-N_4-CHF, (c) NCHF, and (d) CHF electrodes operated under CO_2 and Ar atmosphere in 0.5 M $KHCO_3$ solution at a scan rate of 10 mV s^{-1} without iR compensation, and (e) comparisons of all electrodes under CO_2 in 0.5 M $KHCO_3$ solution.

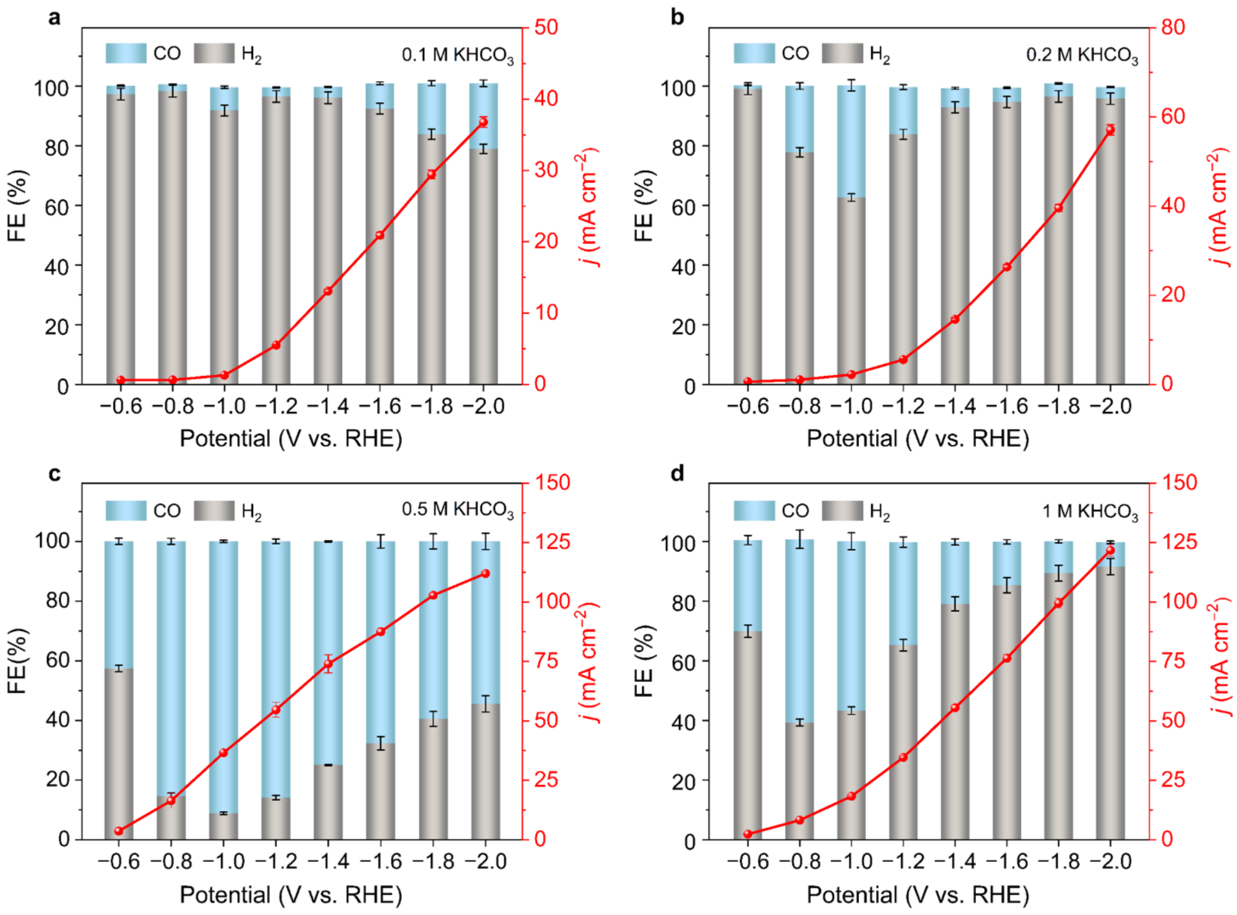

Figure S13. FEs of the ECO_2RR products and current densities on Ni-N_2-CHF at different potentials in (a) 0.1 M $KHCO_3$, (b) 0.2 M $KHCO_3$, (c) 0.5 M $KHCO_3$, and (d) 1 M $KHCO_3$ electrolytes. Error bars were derived from three independent tests.

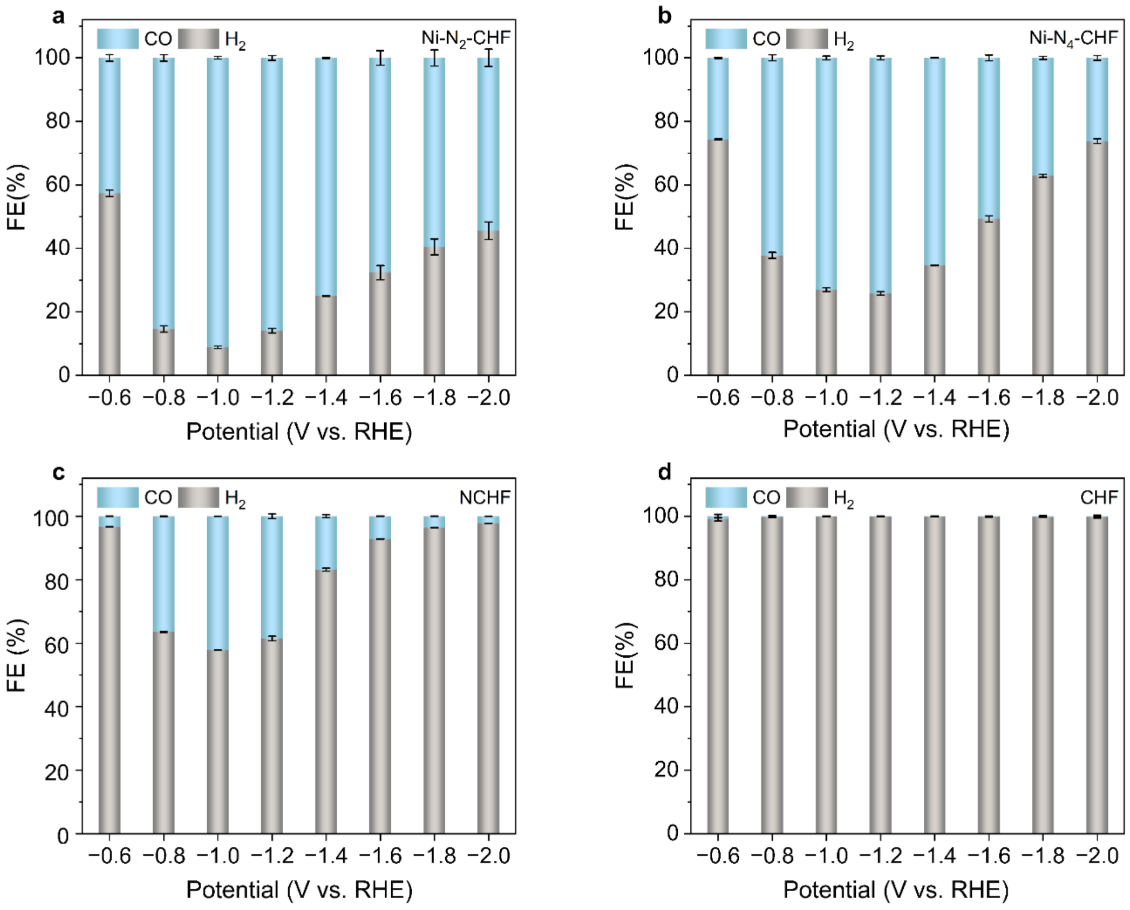

Figure S14. FEs of the ECO_2RR products over (a) Ni-N_2-CHF, (b) Ni-N_4-CHF, (c) NCHF, and (d) CHF electrodes at different potentials in 0.5 M $KHCO_3$ solution. Error bars were derived from three independent tests.

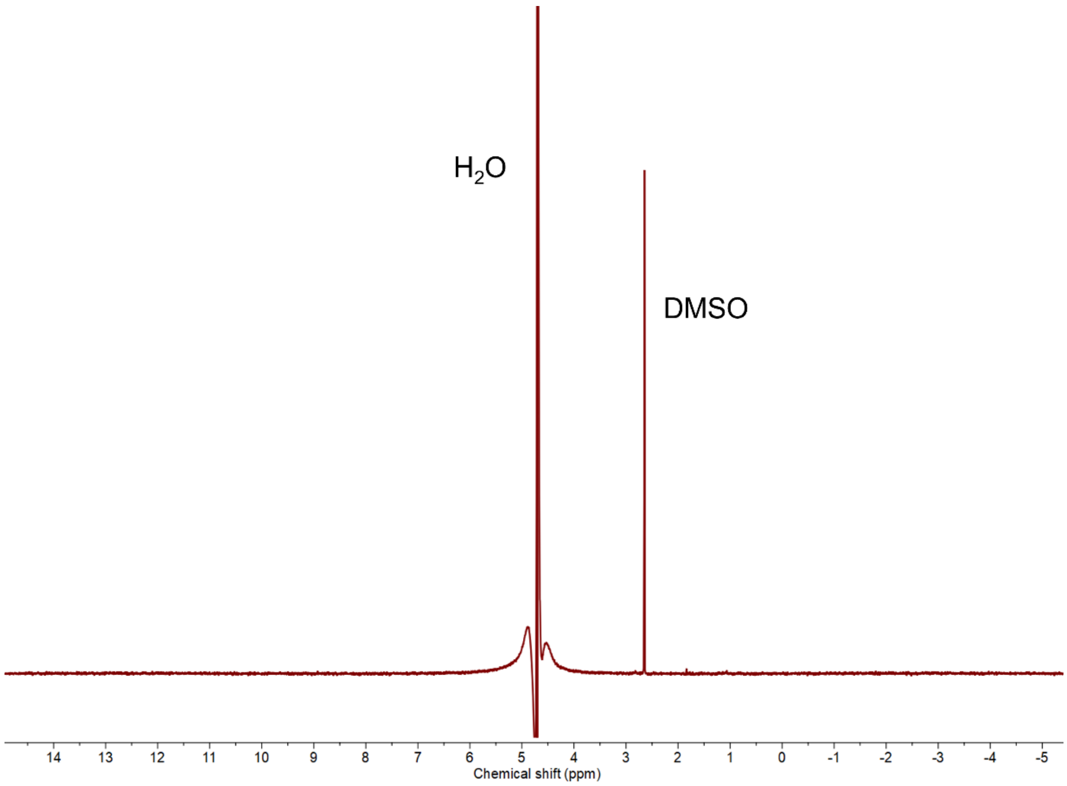

Figure S15. Representative ^{1}H NMR spectrum of the catholyte for Ni-N_2-CHF after 100 h electrolysis at −1.0 V vs. RHE.

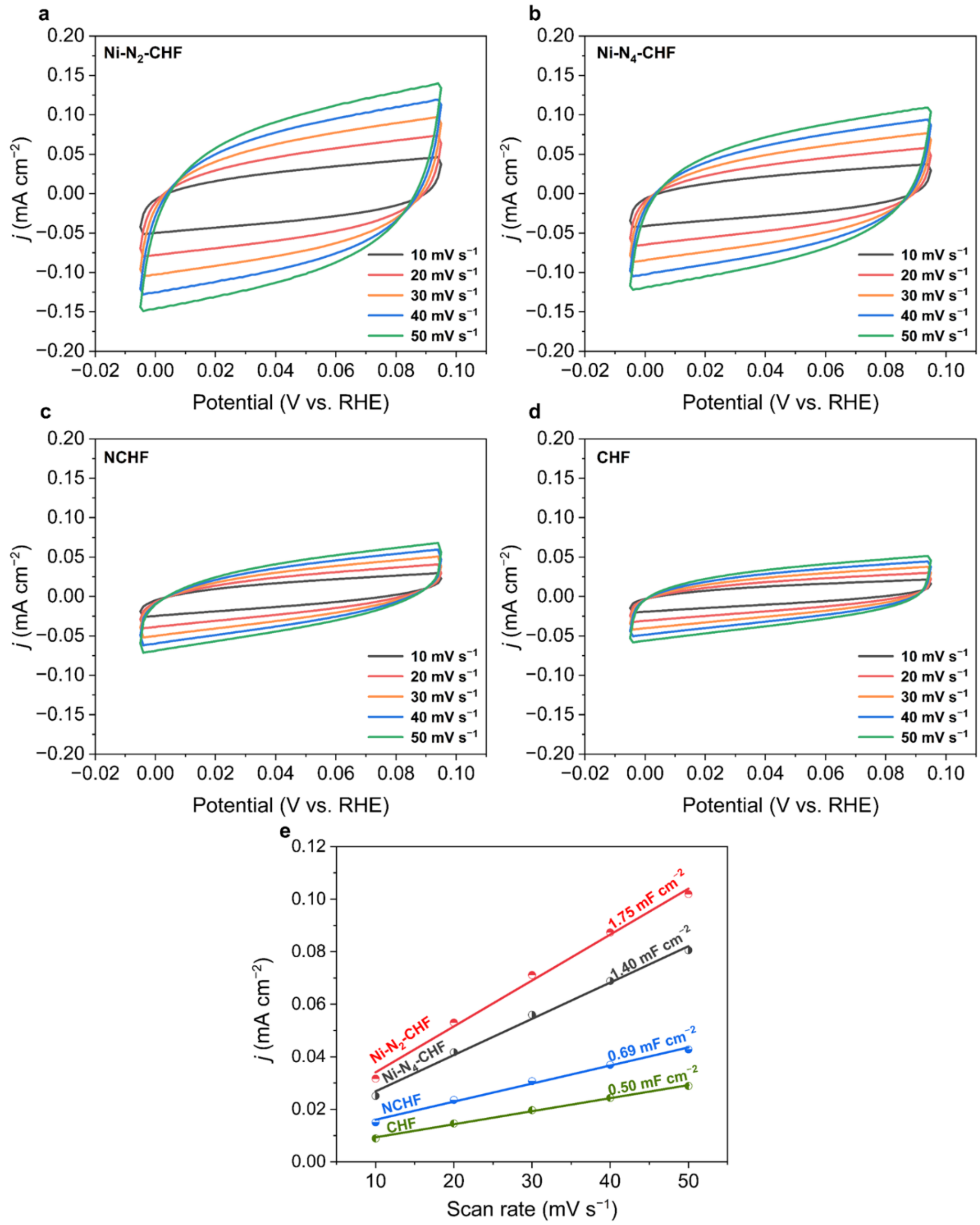

Figure S16. ECSA measurement results. Cyclic voltammetry curves of (a) Ni-N_2-CHF, (b) Ni-N_4-CHF, (c) NCHF, and (d) CHF in Ar-saturated 0.5 M $KHCO_3$. (e) The plots of current density against the scan rate for the above electrodes. The slope of the plot gave the value of C_{dl}.

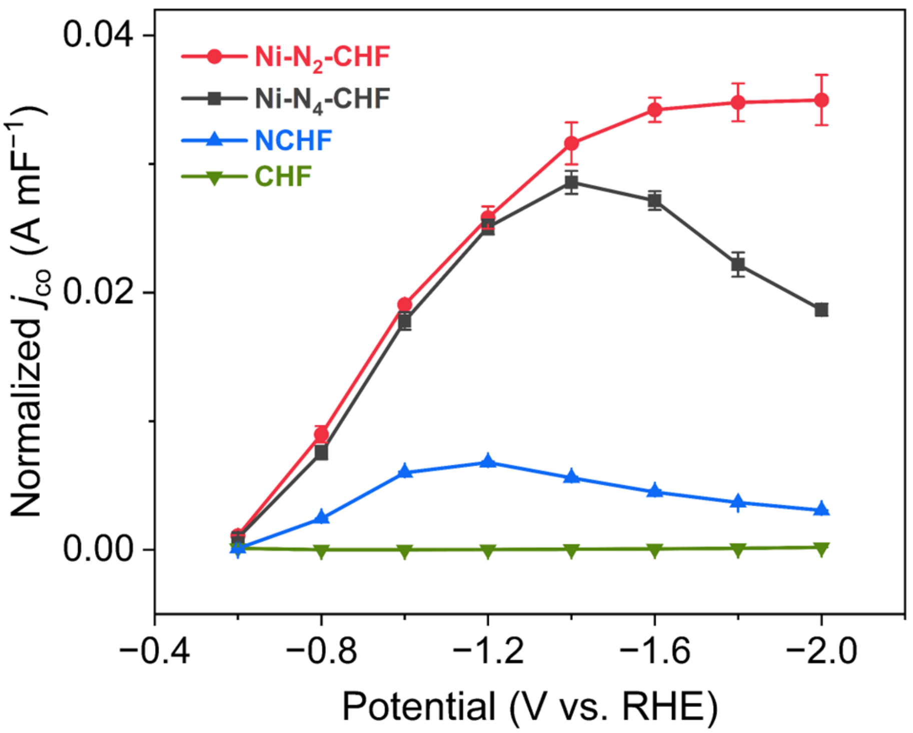

Figure S17. ECSA-normalized partial current density of CO over Ni-N_2-CHF, Ni-N_4-CHF, NCHF, and CHF electrodes.

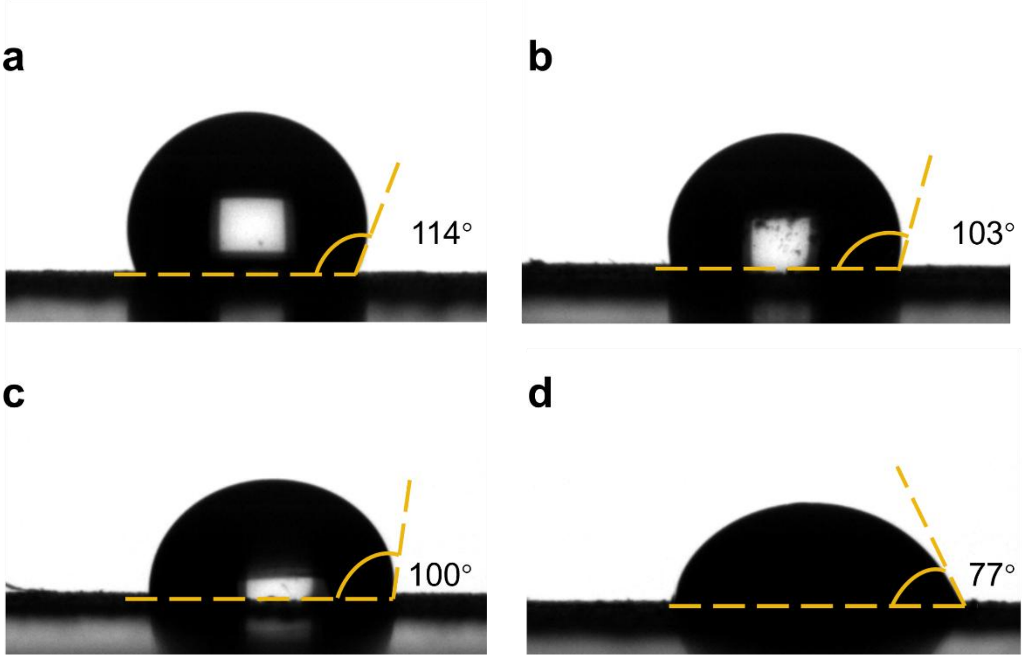

Figure S18. The contact angle measurements on the electrodes of (a) Ni-N_{2}-CHF, (b) Ni-N_{4}-CHF, (c) NCHF, and (d) CHF.

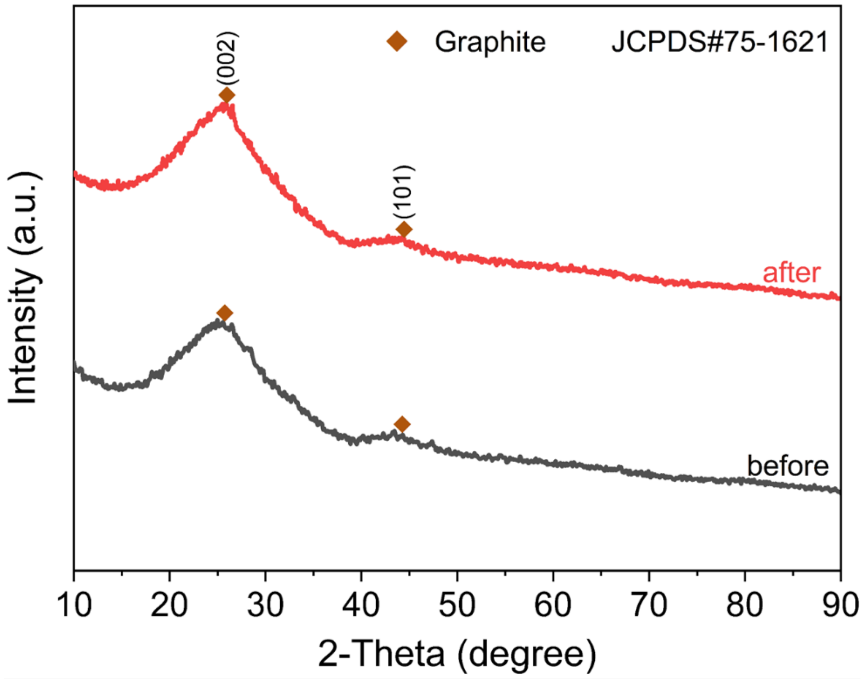

Figure S19. XRD patterns of Ni-N_2-CHF before and after a long-term stability test of 100 h.

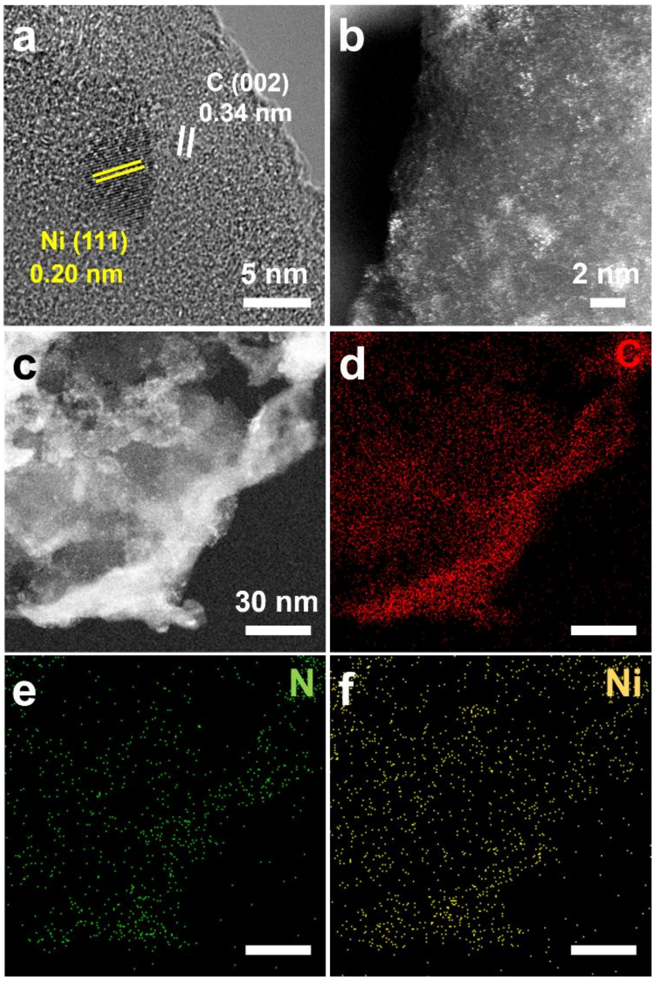

Figure S20. (a) HRTEM, (b) HAADF-STEM image, and (c-f) HAADF-STEM EDS elemental mappings of Ni-N_2-CHF after a long-term stability test of 100 h.

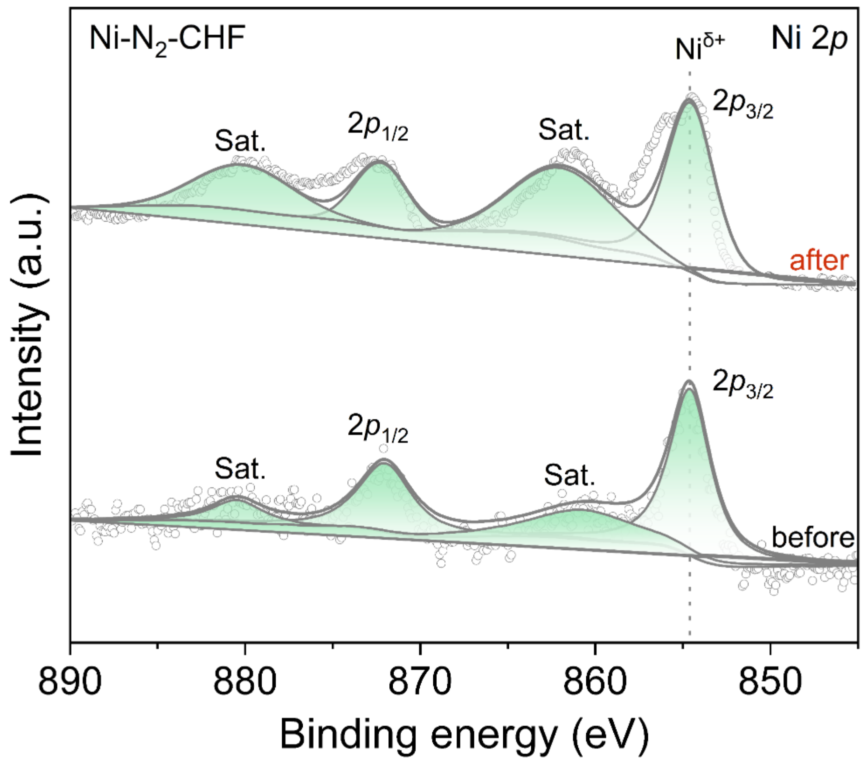

Figure S21. XPS Ni 2p spectra of Ni-N_2-CHF before and after a long-term stability test of 100 h.

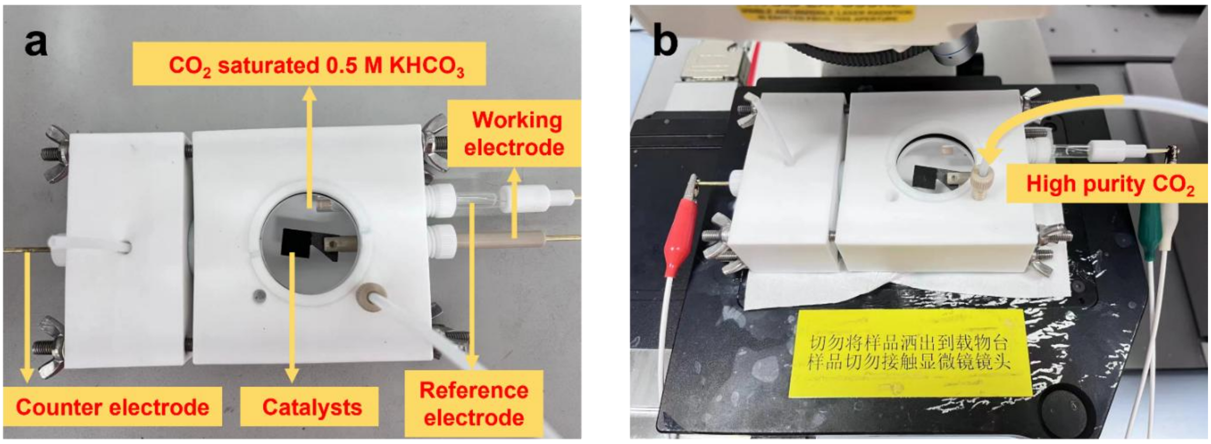

Figure S22. (a) The electrochemical cell, and (b) the corresponding experimental setup for in situ Raman spectroscopy measurement. High-purity CO_2 was continually brought into the electrolyte.

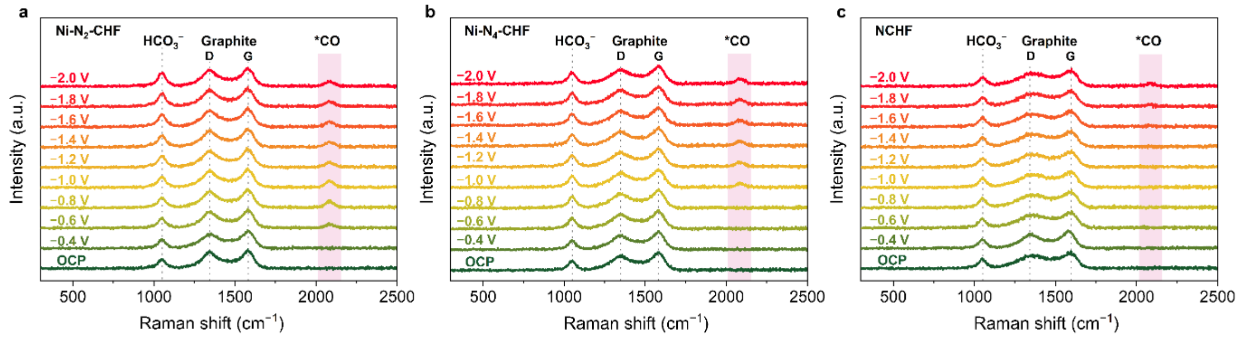

Figure S23. In situ Raman spectra of (a) Ni-N_2-CHF, (b) Ni-N_4-CHF, and (c) NCHF electrodes under ECO_2RR at increasing potentials in CO_2 saturated 0.5 M $KHCO_3$ electrolyte.

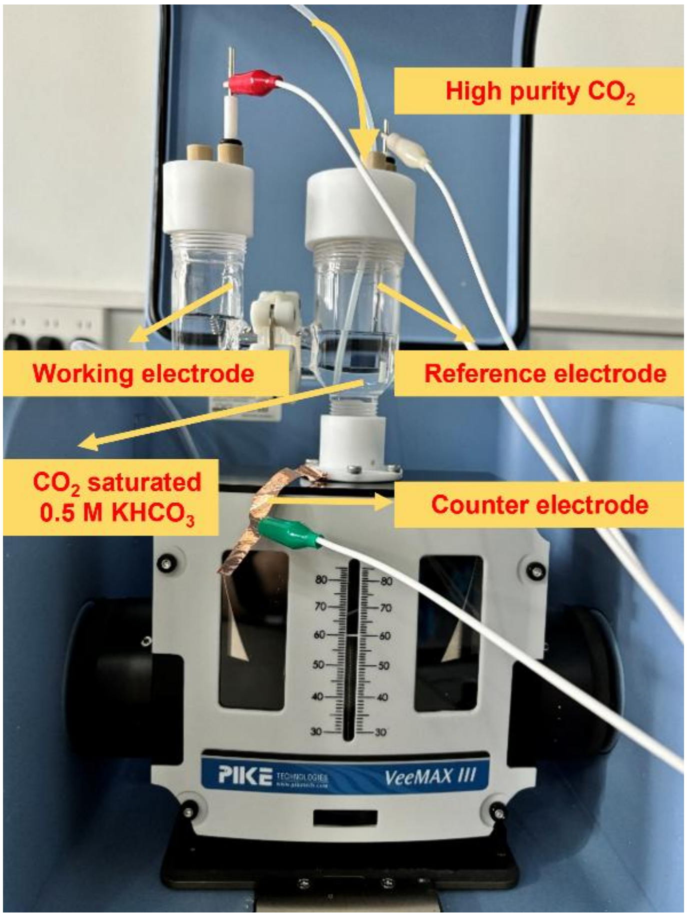

Figure S24. (a) The electrochemical cell, and (b) the corresponding experimental setup for in situ ATR-SEIRAS spectroscopy measurement. High-purity CO_2 was continually brought into the electrolyte.

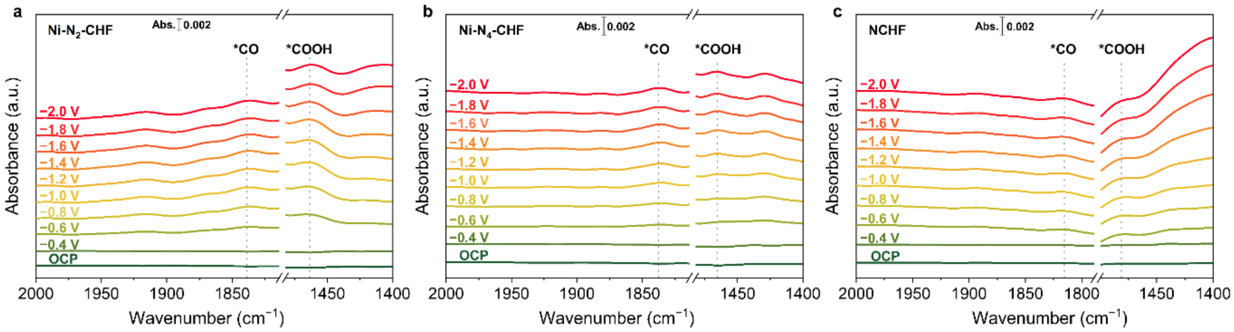

Figure S25. In situ ATR-SEIRAS spectra of (a) Ni-N_2-CHF, (b) Ni-N_4-CHF, and (c) NCHF electrodes under ECO_2RR at increasing potentials in CO_2 saturated 0.5 M $KHCO_3$ electrolyte.

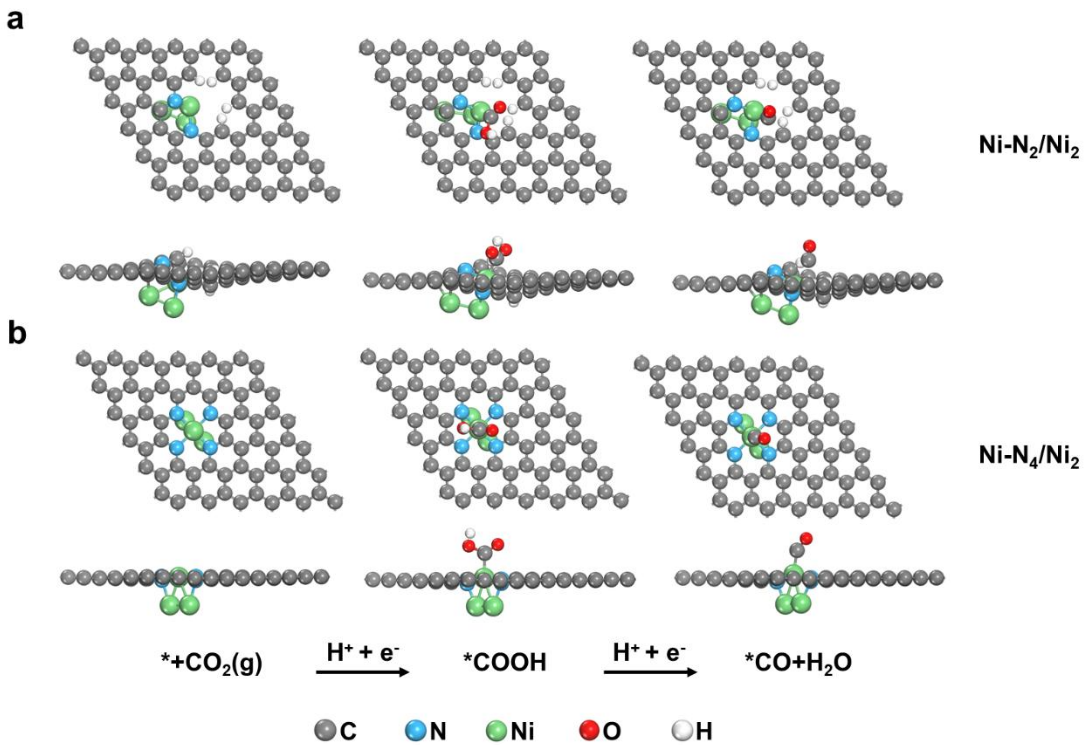

Figure S26. The top and side views of optimized structures of clean surface, *COOH and *CO adsorption on the constructed (a) Ni-N_2/Ni_2 and (b) Ni-N_4/Ni_2 model structures representing Ni-N_2-CHF and Ni-N_4-CHF electrodes, respectively.

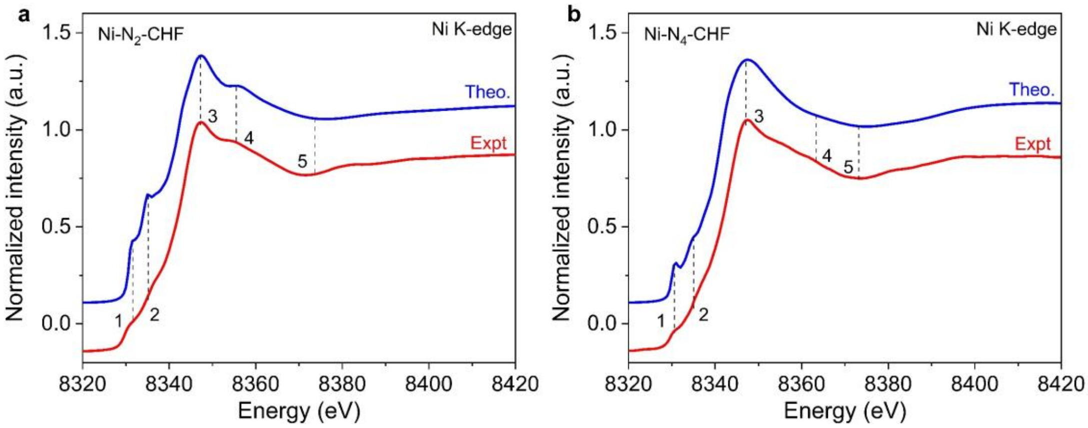

Figure S27. Comparison between the experimental Ni K-edge XANES spectra of Ni-N_2-CHF and Ni-N_4-CHF and the theoretical spectra calculated based on Ni-N_2/Ni_2 and Ni-N_4/Ni_2 models. Some of the main features reproduced are highlighted at points 1-5.

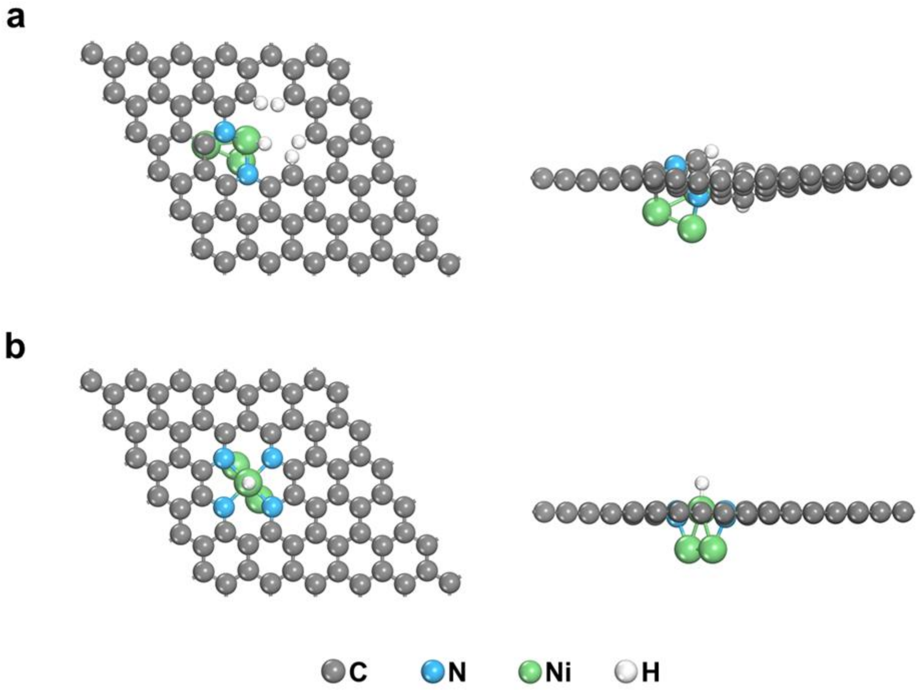

Figure S28. The top and side views of optimized adsorption configurations of *H on (a) Ni-N_2/Ni_2 and (b) Ni-N_4/Ni_2.

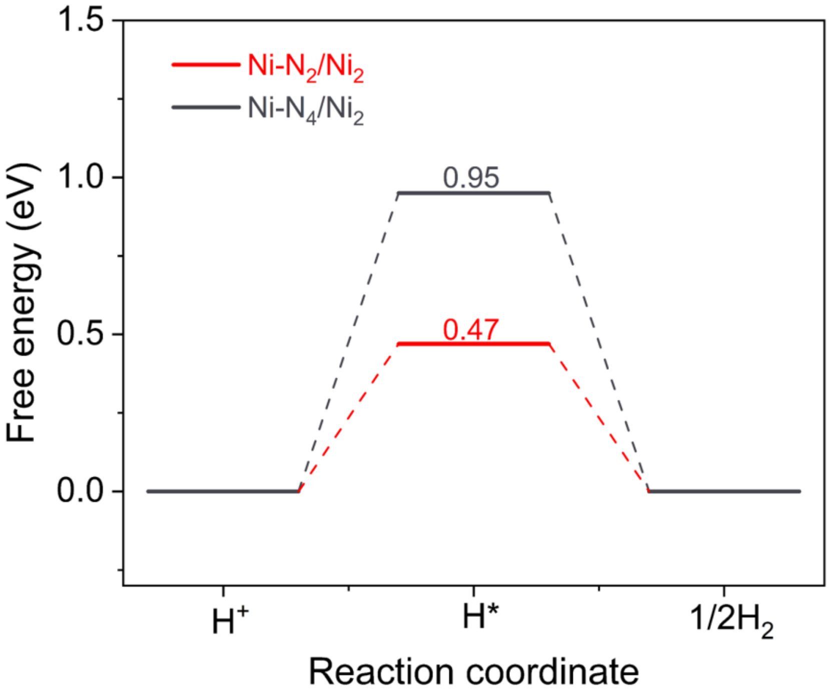

Figure S29. The calculated free-energy diagram for H_2O activation over Ni-N_2-CHF and Ni-N_4-CHF.

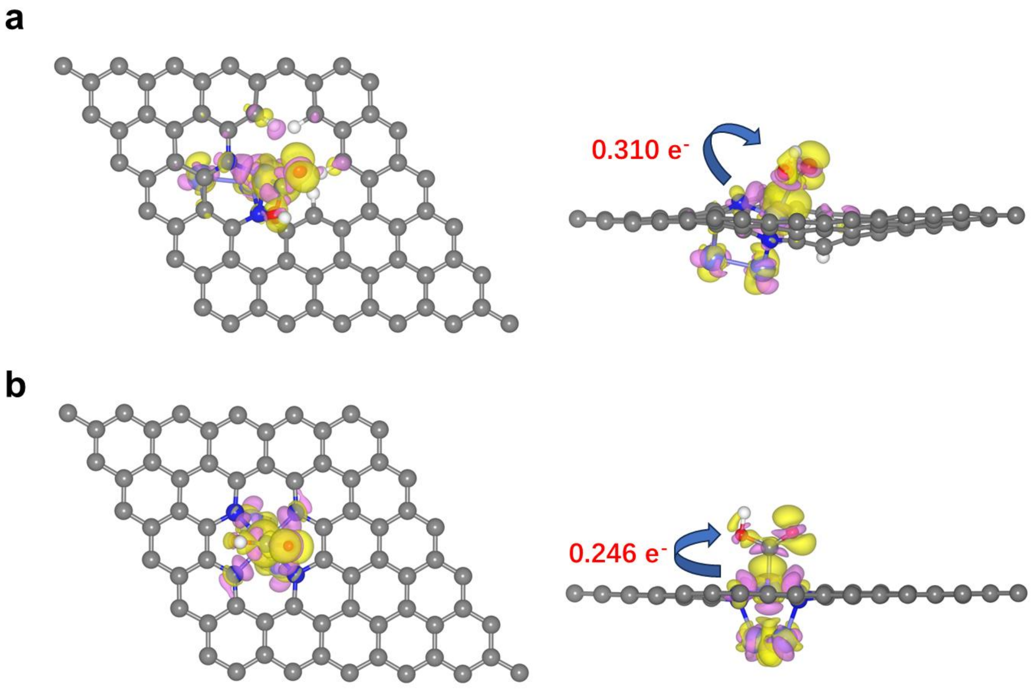

Figure S30. The top and side views of charge difference density analysis of COOH* adsorption on (a) Ni-N_2/Ni_2 and (b) Ni-N_4/Ni_2. The electron accumulation and depletion are represented with yellow and pink contours.

Supporting Tables

Table S1. The weight percentage of Ni in Ni-N_2-CHF and Ni-N_4-CHF determined by inductively coupled plasma optical emission spectroscopy (ICP-OES) measurements.

**Samples**	**Ni-N_2-CHF**	**Ni-N_4-CHF**
Ni content (wt.%)	3.66	3.39

Table S2. The nitrogen content of different types in Ni-N_2-CHF, Ni-N_4-CHF, NCHF, and CHF.

Samples	Pyridinic N (at%)	Ni-N (at%)	Pyrrolic N (at%)	Graphitic N (at%)	Oxidized N (at%)	Total N (at%)
Ni-N_2-CHF	2.31	1.30	2.41	1.01	0.49	7.52
Ni-N_4-CHF	1.10	1.31	0.83	1.91	0.81	5.96
NCHF	3.28	/	2.42	1.50	0.62	7.82
CHF	1.17	/	0.84	3.18	0.83	6.02

Table S3. The average valence states of the Ni species in Ni-N_2-CHF and Ni-N_4-CHF with Ni foil and NiPc as references. (The Edge energy is obtained by taking a first-order derivative of X_μ (E) in the XANES spectrum, equal to the horizontal coordinate value corresponding to the maximum value of the first-order derivate. X_μ (E): absorption coefficient).

**Samples**	**Ni foil**	**Ni-N_2-CHF**	**Ni-N_4-CHF**	**NiPc**
Edge energy (eV)	8333.00	8336.15	8338.32	8347.20
Valence states	0.00	+0.46	+0.78	+2.08

Table S4. Structural parameters obtained from the EXAFS fitting on Ni-N_2-CHF and Ni-N_4-CHF with Ni foil, NiO and NiPc as references. (N: coordination number; R: bond distance; σ^2: Debye-Waller factors; ΔE_0: the inner potential correction).

Samples	Coordination	N	R (Å)	ΔE_0 (eV)	σ^2 (10^{-3}Å^2)	R factor
Ni-N_2-CHF	Ni-N	1.9±0.4	1.96±0.02	5.6±1.8	6.0±1.3	0.01
	Ni-Ni	2.3±0.3	2.49±0.02	5.2±0.9		
Ni-N_4-CHF	Ni-N	3.9±0.8	2.02±0.02	8.2±4.1	0.2±2.9	0.02
	Ni-Ni	2.4±0.6	2.53±0.02	5.6±4.2		
Ni foil	Ni-Ni	12	2.49	7.3±1.4	6.8±2.2	0.01
NiO	Ni-O	6	2.11	6.2±2.4	7.2±1.4	0.02
	Ni-Ni	12	2.98		5.1±2.8	
NiPc	Ni-N	4.0±0.7	1.90±0.02	5.3±2.1	3.7±1.4	0.02
	Ni-C	4.3±1.0	2.90±0.02		7.7±2.2	

Table S5. The spin numbers of the electron paramagnetic resonance (EPR) spectra of Ni-N_2-CHF, Ni-N_4-CHF, NCHF, and CHF.

**Samples**	**Ni-N_2-CHF**	**Ni-N_4-CHF**	**NCHF**	**CHF**
Spin number /10^{12} mg^{-1}	2.79	1.13	2.93	1.81

Table S6. Comparison of the ECO_2RR performance for the sample in this work to previously reported carbon supporting electrodes with Ni-N-C sites for CO production.

Electrodes	Potential (V vs. RHE)	FE_{CO} (%)	Stability (h)	Electrolyte	Ref.
$Ni-N_2-CHF$	−1.0	91.0	100	0.5 M $KHCO_3$	This work
NiSAs@3D-INCT	−1.0	91.4	12	0.5 M $KHCO_3$	[10]
NiSA-NGA	−0.8	90.2	6	0.5 M $KHCO_3$	[11]
NC-CNTs (Ni)	−0.8	90.0	10	0.1 M $KHCO_3$	[12]
Ni-NC (HPU)	−0.8	91.0	27	0.5 M $KHCO_3$	[13]
$Ni-N_3-C$	−0.65	95.6	10	0.5 M $KHCO_3$	[14]
$Ni_2-N_5C_2$	−0.88	90.1	/	0.5 M $KHCO_3$	[15]
NiSAs/N-C	−1.0	70.3	60	0.5 M $KHCO_3$	[16]
ACP/S-N-Ni	−0.77	91.0	14	0.5 M $KHCO_3$	[17]
SE-NiSAs@PNC	−1.0	88.0	60	0.5 M $KHCO_3$	[18]
NiSA-N-CNTs	−0.7	91.3	/	0.5 M $KHCO_3$	[19]

Table S7. Bader charge of Ni atom in Ni-N_2/Ni_2 and Ni-N_4/Ni_2.

**Catalysts**	**Ni-N_2/Ni_2**	**Ni-N_4/Ni_2**
Bader charge	+0.51	+0.81

References

[1] H. Yasuda, J. T. Tsai, *J. Appl. Polym. Sci.* **1974**, *18*, 805-819**.**
[2] S. Nago, Y. Mizutani, *J. Membr. Sci.* **1996**, *116*, 1-7.
[3] G. Kresse, J. Hafner, *Phys. Rev. B Condens Matter.* **1993**, *48*, 13115-13118.
[4] G. Kresse, J. Furthmüller, *Comput. Mater. Sci.* **1996**, *6*, 15-50.
[5] G. Kresse, J. Furthmüller, *Phys. Rev. B Condens.* **1996**, *54*, 11169-11186.
[6] P. E. Blöchl, *Phys. Rev. B Condens.* **1994**, *50*, 17953-17979.
[7] J. P. Perdew, A.Ruzsinszky, G. I.Csonka, O. A.Vydrov, G. E. Scuseria, L. A. Constantin, *Phys. Rev. Lett.* **2008**, *100*, 136406.
[8] M. Ropo, K. kokko, L.Vitos, *Phys. Rev. B* **2008**, *77*, 195445.
[9] S. Grimme, S. Ehrlich, L. Goerigk, *J. Comput. Chem.* **2011**, *32*, 1456-1465.
[10] K. Wang, B. Chen, Y. Xuan, W. Fan, N. Sun, S. Chang, G. Meng, *Appl. Catal. B Environ.* **2023**, *338*, 123083.
[11] K. W. Mou, Z. P. Chen, X. P. Zhang, M. Y. Jiao, X. X. Zhang, X. Ge, W. Zhang, L. C. Liu, *Small* **2019**, *15*, 1903668.
[12] Q. Fan, P. Hou, C. Choi, T.-S. Wu, S. Hong, F. Li, Y.-L. Soo, P. Kang, Y. Jung, Z. Sun, *Adv. Energy Mater.* **2020**, *10*, 1903068.
[13] Y. Li, X. F. Lu, S. Xi, D. Luan, X. Wang, X. W. D. Lou, *Angew. Chem. Int. Ed.* **2022**, *61*, e202201491.
[14] Y. Zhang, L. Jiao, W. Yang, C. Xie, H.-L. Jiang, *Angew. Chem. Int. Ed.* **2021**, *60*, 7607.
[15] Y.-N. Gong, C.-Y. Cao, W.-J. Shi, J.-H. Zhang, J.-H. Deng, T.-B. Lu, D.-C. Zhong, *Angew. Chem. Int. Ed.* **2022**, *61*, e202215187.
[16] C. Zhang, X. Dai, T. Yao, W. Chen, X. Wang, J. Wang, J. Yang, J. Yang, S. Wei, Y. Wu, Y. Li, *J. Am. Chem. Soc.* **2017***, 139 (24),* 8078-8081.
[17] S. Li, M. Ceccato, X. Lu, S. Frank, N. Lock, A. Roldan, X.-M. Hu, T. Skrydstrup, K. Daasbjerg, *J. Mater. Chem. A* **2021**, *9*, 1583-1592.
[18] J. Yang, Z. Qiu, C. Zhao, W. Wei, W. Chen, Z. Li, Y. Qu, J. Dong, J. Luo, Z. Li, Y. Wu, *Angew. Chem. Int. Ed.* **2018***, 57,* 14095.
[19] Y. Cheng, S. Zhao, B. Johannessen, J.-P. Veder, M. Saunders, M. R. Rowles, M. Cheng, C. Liu, M. F. Chisholm, R. Marco, H.-M. Cheng, S.-Z. Yang, S. P. Jiang, *Adv. Mater.* **2018**, *30*, 1706287.
